# Novel pathway for the sonochemical synthesis of silver nanoparticles with near-spherical shape and high stability in aqueous media

**DOI:** 10.1038/s41598-022-04921-9

**Published:** 2022-01-18

**Authors:** Bryan Calderón-Jiménez, Antonio R. Montoro Bustos, Reinaldo Pereira Reyes, Sergio A. Paniagua, José R. Vega-Baudrit

**Affiliations:** 1 Chemical Metrology Division, National Metrology Laboratory of Costa Rica (LCM), San José, 11501-2060 Costa Rica; 2 National Laboratory of Nanotechnology, National Center of High Technology, San José, 1174-1200 Costa Rica; 3 Ph.D Program in Natural Science for Development (DOCINADE), Technological Institute of Costa Rica, National University, State Distance University, San José, 159-7050 Costa Rica; 4 Material Measurement Laboratory, Chemical Sciences Division, National Institute of Standards and Technology, Gaithersburg, MD 20899 USA

**Keywords:** Nanoparticles, Nanoparticle synthesis

## Abstract

The present study shows the development of a novel sonochemical synthesis pathway of sub-15 nm silver nanoparticles (AgNPs) with quasi-spherical shape and high stability in aqueous suspension. Different analytical techniques such as on-line UV–Vis spectroscopy, Atomic Force Microscopy (AFM), and Transmission Electron Microscopy (TEM) were complementarily used to characterize the evolution of the properties of AgNPs synthesized with this new route. Furthermore, different centrifugation conditions were studied to establish a practical, simple and straightforward purification method. Particle size was determined by TEM employing two different deposition methods, showing that purified AgNPs have a size of 8.1 nm ± 2.4 nm with a narrow dispersion of the size distribution (95% coverage interval from 3.4 to 13 nm). Critical information of the shape and crystalline structure of these sub-15 nm AgNPs, provided by shape descriptors (circularity and roundness) using TEM and high resolution (HR)-TEM measurements, confirmed the generation of AgNPs with quasi-spherical shapes with certain twin-fault particles promoted by the high energy of the ultrasonic treatment. Elemental analysis by TEM-EDS confirmed the high purity of the sub-15 nm AgNPs, consisting solely of Ag. At the optical level, these AgNPs showed a bandgap energy of (2.795 ± 0.002) eV. Finally, the evaluation of the effects of ultraviolet radiation (UVC: 254 nm and UVA: 365 nm) and storage temperature on the spectral stability revealed high stability of the optical properties and subsequently dimensional properties of sub-15 nm AgNPs in the short-term (600 min) and long-term (24 weeks).

## Introduction

In recent years the world has seen an exponential growth of applications and technologies developed in the field of nanoscience and nanotechnology. Mainly, silver nanoparticles (AgNPs) currently drive a predominant amount of new scientific developments and innovation, ranging from the generation of new antiseptic, antifungal and virucidal agents, catalysts, food packaging, textiles, photoelectronics, sensors, bioengineering, biomedicine, electrochemistry, heterogeneous catalysis, environmental treatment systems among others^1^.

Most of these applications benefit from the intrinsic physicochemical properties that AgNPs exhibit at the nanoscale: where particle size^2^, size distribution^3^ and shape^4^ are the predominant dimensional properties to establish the design and type of application of these nanomaterials. An imperative need to improve the applicability of AgNPs in new advanced technologies in the field of plasmonic devices^5^ and nanoelectronic devices construction^6^, as well as to develop new applications of medical devices and biomedical therapies for relevant diseases such as certain types of cancer^7^, virus infections^8^ and pathogens, among others.

In this context, finding new routes for the synthesis of AgNPs that increase the stability of their dimensional properties is an indispensable scientific task for the development of advanced applications in the field of nanoscience, nanotechnology, and nanometrology^9^. In fact, recent efforts have focused on the development of bottom-up wet synthesis routes to obtain AgNPs with high size control^10^, monodispersed size distribution^11^, and highly spherical shape^12^. Among them, several routes for the synthesis of AgNPs have been reported for the obtention of different particle sizes ranging from 5 to 100 nm^13^ and even larger than 100 nm^14^. Interestingly, it has been demonstrated that some antibacterial^13^, optical^15^, and catalytic^16^ properties of AgNPs are significantly enhanced for particle sizes smaller than 15 nm (sub-15 nm). Thus, despite the high surface area and tendency to agglomeration, aggregation, and/or dissolution processes^17^, the obtention of highly stable sub-15 nm AgNPs under controlled conditions has attracted great interest from the scientific community.

However, despite the long variety of approaches for the synthesis of AgNPs^18^, the routes that achieve short-term stability in aqueous suspension are limited^19^, and particularly scarce for the production of AgNPs with high long-term stability. Therefore, there is a clear need to develop new synthesis routes for sub-15 nm AgNPs with high stability (> 3 months), to maintain their optical properties and subsequently their dimensional properties, as well as to promote the development of advanced applications with high stability requirements (e.g.; reference materials at the nanoscale).

The production and obtention of NPs and nanostructured materials using high-intensity ultrasonic radiation has been studied during the last decades^20^. Specifically, the sonochemical method has been studied for the synthesis of different types of noble metal NPs such as Au, Ag, Pt, Pb^21^, and other nanomaterials such as alloys, oxides, composites, among others^22^.

The fundamental mechanism of sonochemical synthesis of metallic NPs is the cavitation phenomenon. This hydrodynamic effect generates gaseous bubbles with µm diameters that collapse and implode within the medium, generating sufficient energy, temperature (> 5000 K) and pressure (~ 1800 atm) to convert the solvent molecules^23^ in most cases water, into free radicals of highly reactive species (HO_2_^**·**^, H^**·**^, OH^**·**^, and perhaps e_aq_^−^ (solvated electron)). These are capable of providing sufficient chemical potential to reduce noble metals, among which Ag stands out^23^.

The main advantages of sonochemical methods include fast reaction speed, controllable reaction conditions, simplicity and safety of the technique, obtention of spherical and uniform shapes, distributions with a certain symmetry, and with a high purity to the nanomaterials^24^. Furthermore, the sonochemical method differs from the NP wet synthesis methods by not requiring relative high temperatures to promote the synthesis reaction^25^, long reaction times^26^, pH control of the NP suspension^27^, use of shape control agents (e.g. tannic acid)^28^, and use of steric capping agents to control shape and stability of NPs^29^. More details on the different conditions necessary to generate monodispersed and quasi-spherical AgNPs using classical methods are summarized in previous studies^1^. Also, the use of sonochemistry offers the remarkable possibility of generating AgNPs with particular small particle sizes^30^ which places it as a promising pathway to obtain AgNPs in the range of sub-15 nm with defined dimensional properties (size, size distribution, and shape) and potential stable metallic nuclei in aqueous media during a long period of time.

This study presents the development of a novel sonochemical synthesis route to obtain sub-15 nm AgNPs. Different analytical techniques such as online UV–Vis spectroscopy, Atomic Force Microscopy (AFM), and Transmission Electron Microscopy (TEM) were complementarily used to characterize the evolution of the properties of AgNPs synthesized with this new route. This multi-technique characterization strategy helped to understand some mechanisms involved in the formation of AgNPs that occur in this new pathway (nucleation, growth, coalescence, agglomeration/aggregation) as well as to design a direct and simple strategy for the purification and adequate characterization of this type of nanomaterial using TEM, HR-TEM, TEM-EDS and UV–Vis. Following this, a thorough evaluation of the short-term stability of sub-15 nm AgNPs under UVC (254 nm) and UVA (365 nm) irradiation conditions as well as their high long-term stability under two different storage temperature conditions has been carried out.

## Results and discussion

### Evolution of AgNPs during sonochemical synthesis.

Understanding the reaction processes and the characterization of the evolution of the bottom-up synthesis of NPs is an arduous and complex task that requires the use of various analytical techniques. Processes such as growth, agglomeration, aggregation, and destabilization of NPs can occur at any stage of the synthesis. Therefore, their study should focus beyond the first few minutes of reaction when reactants are added and reacted. These studies should be long enough to observe the characteristics of the first nuclei or nanocrystals, to study the evolution of their ripening and/or coalescence, growth, as well as to determine the physical and/or chemical properties of the synthesized NPs.

In this context, the sonochemical synthesis route of AgNPs proposed in this research was monitored using multiple analytical techniques over a period of 140 min. To study the evolution of the optical properties of the AgNPs during the sonochemical synthesis, an on-line UV–Vis system (Supplementary Fig. S1a) allowed the measurement of spectrometric scans every 4 min during 140 min. Supplementary Fig S1b,c show the 3D and 2D (contour) plots of the SPR absorption bands generated during the sonochemical irradiation process (first 16 min) and subsequent evolution of the reaction up to 140 min. In these figures it can be observed how in the first minutes of the reaction (0 to 16 min) there was an accelerated increase of the maximum absorbance of the SPR band (Abs_MAX_), indicating that in this first stage of the synthesis the concentration of the AgNPs increased substantially as a function of time (4 to 16 min). This finding can be explained due to the effect of the ultrasonic irradiation that promotes the nucleation and sonocrystallization processes^31^ of NPs due to the presence of highly reactive species such as HO_2_^**·**^, H^**·**^, and OH^**·**^^23^. Moreover, the relatively slow addition (0.60 g min^−1^) of the precursor agent (Ag^+^) to the reaction medium also contributed to the gradual nucleation and growth of AgNPs at the beginning of the sonochemical reaction.

**Figure 1:**
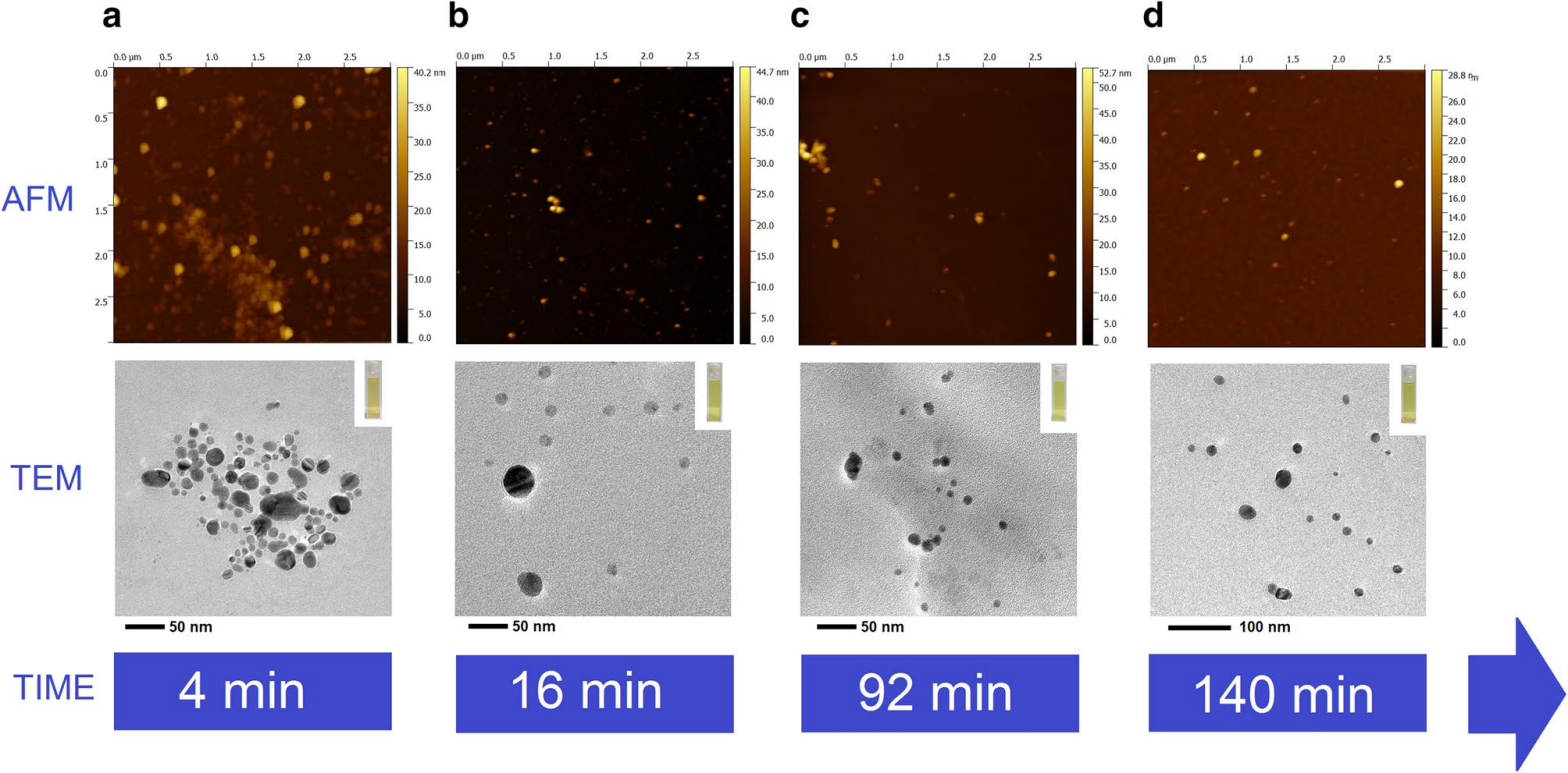
Monitoring the evolution of the sonochemical synthesis of AgNPs using AFM and TEM. (**a**) 4 min, (**b**) 16 min, (**c**) 92 min, (**d**) 140 min.

As shown in Fig. 1, AFM and TEM of the raw samples visually support the evolution of the optical properties of AgNPs during the sonochemical synthesis. A representative AFM micrograph (Fig. 1a) taken in the first 4 min of the sonochemical synthesis corroborated the UV–Vis results. A large amount of AgNPs with particle sizes (median) of ~ 16 nm was observed, with a size dispersion, described by an interquartile range (IQR) of 9.8 nm, indicating a high degree of polydispersity of the AgNPs in these early stages of the synthesis (Supplementary Table S1). An analogous result is observed in the TEM image (Fig. 1a) that confirmed the formation of particle sizes mostly distributed between $\sim$ 2 to $\sim$ 20 nm. The TEM images also showed the presence of a low proportion of AgNPs with particle sizes > 20 nm, as well as the formation of AgNPs with non-spherical ovoid-like shapes, which may be originated from the melting of two different nucleus during the sonochemical process^23^, Ostwald ripening processes^32^ or coalescence^33^ of multiple NPs, typically observed in the early stages of nucleation and growth of AgNPs in sonochemical synthesis.

Once this first stage of the synthesis was completed (4 min) but before the sonochemical treatment process was finished (~ 16 min, Fig. 1b), 10 mL of a 2 mM NaCit solution was introduced into the reaction system. The purpose of the addition of citrate anions (Cit^−^) to the synthesis medium was to prevent the destabilization of the AgNPs. It is important to highlight that UV–Vis monitoring studies of AgNPs sonochemical syntheses^17^ reported that BH_4_^−^ ion is considered a labile ligand unable to prevent the destabilization of AgNPs for periods longer than 80 min. For that reason, BH_4_^−^ was selected here as a strong reducing agent^34^ to help the ultrasonic irradiation in promoting rapid nucleation and formation of the first nanocrystals within the reaction system. Subsequently, once a high concentration of nanocrystals was generated, Cit^-^ electrostatically stabilized the surface of the metal nucleus, while degradation of BH_4_^−^ ions by the action of H_2_O present in the reaction medium occurred^13,17^.

After finishing the addition of the precursor agent (Ag^+^) and the sonochemical treatment, it can be observed in Supplementary Fig S1c that the colloidal system adopts a metastable zone or plateau that goes from 16 min to approximately 80 min. In this plateau the optical properties of AgNPs such as maximum SPR wavelength (${\lambda }_{SPR}$), maximum absorbance (Abs_MAX_) and full width at half maximum (FWHM) remain unchanged as the post-synthesis time elapses. This result suggests that from 16 to 80 min the colloidal system remained highly reproducible, with low formation of agglomerations or aggregations, and with minimal changes in particle size, concentration, and polydispersity of AgNP size distribution. The evolution of AgNPs at the end of the sonochemical treatment was evident in Fig. 1b, where the AFM micrograph showed a lower polydispersity (IQR 5.7 nm) and a particle size (median) of 6.4 nm of AgNP population (Supplementary Table S1). More detailed monitoring of the evolution of the aggregation and agglomeration processes during the sonochemical synthesis by AFM was included in Supplementary Fig S2. TEM micrographs further confirmed the presence of AgNPs with small particle sizes, as well as the presence of a small portion of NPs with particle sizes > 20 nm, that seemed even larger than those observed at the beginning of the sonochemical reaction (Fig. 1a). This observed evolution in the mean particle size can be explained due to digestive ripening processes that together with the addition of Cit^−^ in the last minutes of the sonochemical treatment promoted the production of AgNPs smaller than those observed at the beginning of the sonochemical synthesis.

At a reaction time of 80 min, the optical properties of AgNPs changed showing a slight decrease in Abs_MAX_ (Supplementary Fig. S1b), that corresponded to a minimal change in the particle concentration, and a redshift of ${\lambda }_{SPR}$ (Supplementary Fig. S1c) associated with an increase in the particle size. The trend in the optical properties of AgNPs was corroborated by AFM micrographs (Fig. 1c and Supplementary Fig. S3) at 92 min, that identified an increase in the particle size of the synthesized AgNPs up to ~ 9.8 nm (Supplementary Table S1). Besides, the generation of a small portion of AgNPs with particle sizes mostly between 15 and 40 nm was also detected by AFM (Supplementary Fig. S3 and Supplementary Table S1). AFM also revealed the presence of a small amount of big agglomerates/aggregates larger than 20 nm in the final stages of the evolution of the synthesis reaction (Supplementary Fig. S2). This finding correlated with the destabilization and oxidation of some metallic nucleus of AgNPs produced by the effect of the decomposition of BH_4_^-^when reacting with water that increased the aggregation and agglomeration (Supplementary Fig. S2). The addition of Cit^−^ to the reaction medium, previously described, significantly reduced the formation of agglomerates and aggregates compared to other synthesis routes^49^. The high energy released by cavitation bubbles from the ultrasonic irradiation may also contribute to the formation of AgNPs larger than 15 nm through the melting of two different nuclei and to the creation of aggregates with complex nanostructures. This aspect will be discussed in more detail in the further section.

Finally, 140 min after the beginning of the sonochemical synthesis the colloidal system found a new equilibrium (Supplementary Fig. S1b,c), producing sub-15 nm AgNPs with a median particle size, measured by AFM, of ~ 9.1 nm and an IQR of 2.1 nm (Fig. 1 and Supplementary Table S1). In this stage, the presence of big agglomerates and aggregates of AgNPs was also found (Supplementary Fig. S2). The synthesis batch of AgNP suspension (raw AgNPs) was stored under controlled conditions for further characterization, that will be outlined in the following section.

### Dimensional properties of raw AgNP synthesis batch material and assessment of the purification by centrifugation

**Figure 2:**
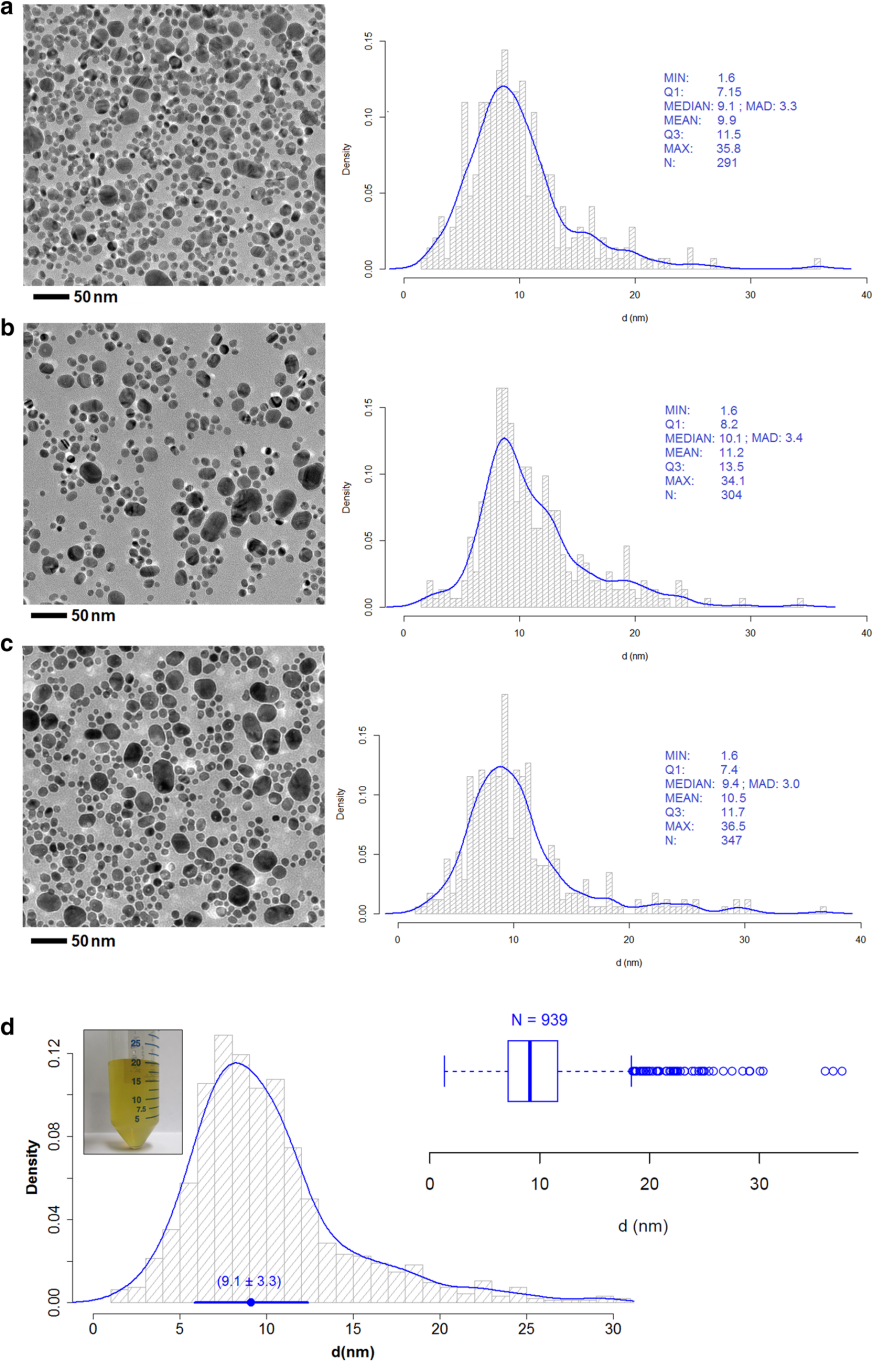
Particle size and particle size distribution of the raw AgNPs (**a**–**c**) TEM microscopies taken in different areas of the grid, (**d**) Particle size and size distribution of the raw AgNPs (overall result).

TEM analysis of the crude AgNP synthesis batch were carried out at different locations of the grid to achieve representative measurements of the dimensional properties of the crude material obtained in the synthesis. The results in Fig. 2a–c showed that the raw AgNP suspension exhibited a high degree of homogeneity of the particle size. Particularly, TEM micrographs of crude AgNPs taken at three different locations showed particle sizes (Median) of 9.1 nm, 10.1 nm, and 9.4 nm, respectively. Moreover, very similar values for the dispersion of particle sizes, expressed as the median absolute deviation (MAD), of 3.3 nm, 3.4 nm, and 3.0 nm, respectively were obtained. Additionally, the particle size distributions of the three different locations (based on a Kernel distribution) showed a very similar trend; they did not significantly differ statistically at 95% confidence (F_test_ < F_crit_) that made it possible to combine the individual particles from the three different locations to estimate an overall value of the particle size and size distribution of raw AgNPs, displayed in Fig. 2d.

Overall, the TEM characterization of the raw material provided a very approximate estimate of the particle size (9.1 ± 3.3) nm and revealed the existence of a narrow size distribution (IQR 4.5 nm). While a positive asymmetry yielded by the presence of a small portion (13%) of AgNPs > 15 nm, produced during the synthetic process, was detected in the global particle size distribution, the majority of AgNPs ($\sim$ 87%) was distributed around the median ± MAD. In this context, the measurements made by AFM at the end of the monitoring (140 min) show an even narrower IQR (2.1 nm) than that obtained in TEM characterization. This variation can be attributed to the already demonstrated method dependency of these measurements have on the nano-scale^9^.

Another critical aspect observed during the characterization of the raw AgNPs was the formation of a small brown sediment after storing the material at a temperature of (4 ± 2) °C for 24 h. While, the sediment was easily redispersed after 2 min of manual shaking or vortexing, it appeared at the bottom of the vessel’s after prolonged storage time. Note that despite the presence of the brown sediment, the raw material maintained an intense yellow color characteristic of AgNPs of small particle size (~ 9 nm). All of the above indicates that the stability of the raw material had not been significantly impacted during these first hours post-synthesis and that possibly the sediment corresponds to byproducts of the sonochemical synthesis that due to their larger size tend to sediment quickly as reported in other AgNPs synthesis studies^14^.

To gain a deeper insight, the sediment was analyzed by TEM (Supplementary Fig. S4), which showed the presence of extremely large aggregates (> 100 nm) constituted by several AgNPs of sizes between 30 to 50 nm linked by nano-bridges coming from the melting/co-growth of AgNPs. Comparison between TEM analysis of the sediment and AFM micrographs during the evolution of the sonochemical synthesis (Supplementary Fig. S2) demonstrated that these aggregates were generated in the first minutes (4 min and 16 min) of the sonochemical synthesis, which confirmed that they were formed as secondary products during the ultrasonic treatment. It is likely that the shock waves generated by the ultrasonic treatment promoted the collision between close AgNPs, which if they have an appropriate angle and sufficient speed can induce their melting^35^, that will result in the generation of these gross impurities or secondary products.

To eliminate the presence of these impurities, centrifugation was selected as a simple, fast, and direct technique for the purification of raw AgNPs. Benefits of the centrifugation include high efficiency, scalable production, and generation of aggregation-free NPs suspension^36^. However, there is currently a lack of harmonization or consensus on the experimental conditions for the centrifugation and purification of AgNPs (time, speed, time, temperature), mainly because of the varying intrinsic properties of AgNPs such as particle size, viscosity of the medium, and the content of impurities, among others, that makes necessary a customized optimization of the purification procedure. Based on existing literature^37,38^, the purification of raw AgNP synthesis batch was carried out for 20 min at 4 °C using three different RCFs, 10,000*g*, 20,000*g*, and 30,000*g*.

**Figure 3:**
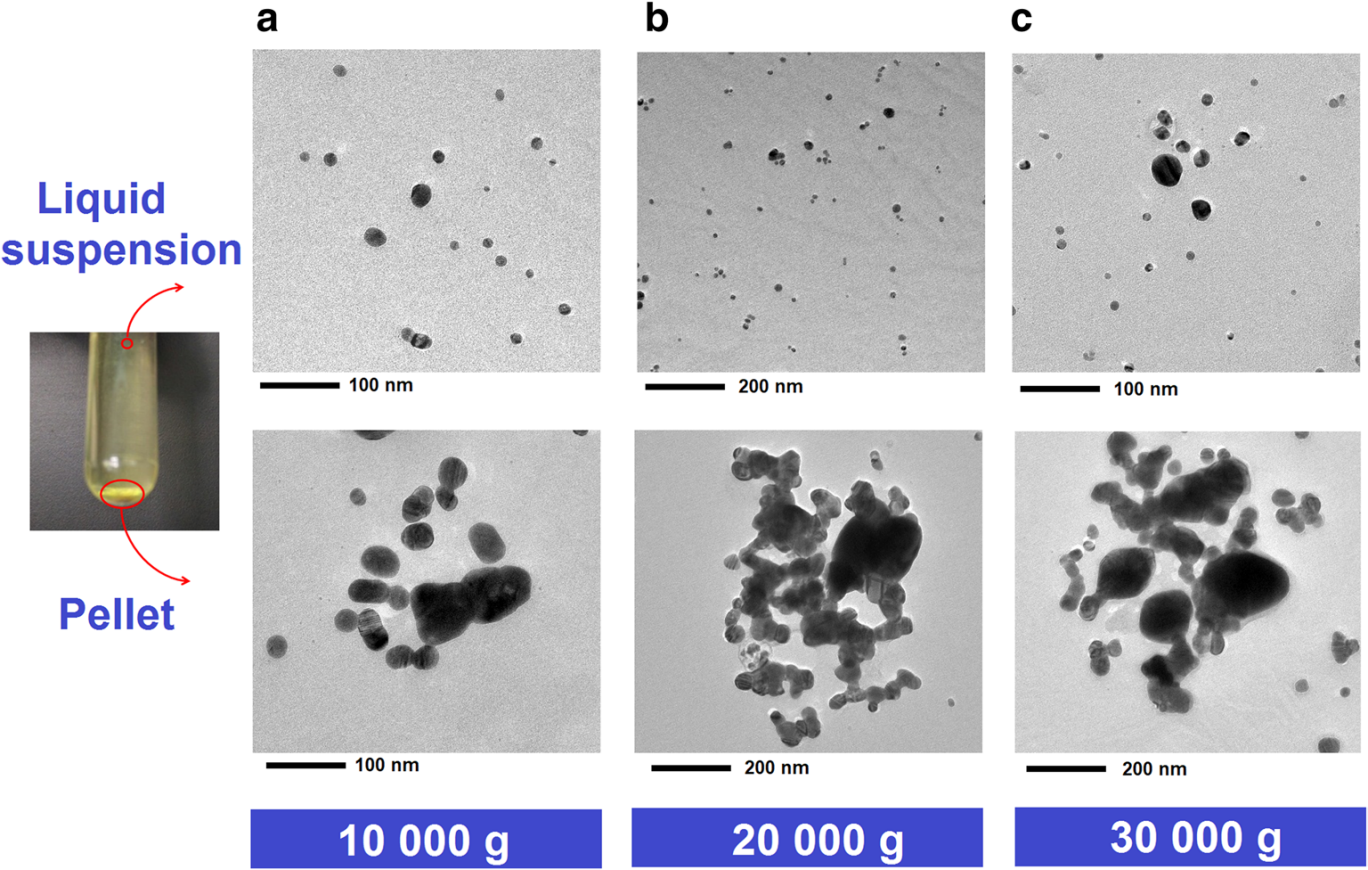
TEM images from the liquid suspension (supernatant) and pellet (sediment) obtained after purification of the raw AgNPs using different relative centrifugal fields.

Figure 3 shows the results obtained from the purification process of the raw AgNPs. The centrifugation at 10,000*g* succeeded in separating AgNPs larger than 30 nm present in the raw batch (Fig. 3a). In contrast, centrifugation at 20,000*g* (Fig. 3b) and 30,000*g* (Fig. 3c) led to a higher sedimentation of AgNPs larger than 200 nm, complex aggregates, and AgNPs with a medium particle size ($>$ 30 nm). Furthermore, these results suggested that there was no substantial difference between the two accelerations (20,000*g*, and 30,000*g*), so that centrifugation at 30,000*g* could be the most conservative alternative to ensure and promote higher sedimentation of impurities from sonochemical synthesis. Finally, representative TEM micrographs of the supernatants (Fig. 3), showed that a larger portion of sub-15 nm AgNPs remained in suspension for centrifugation at 20,000*g* and 30,000*g*. Therefore, centrifugation at 30,000*g* during 20 min at 4 °C was selected for the separation, sedimentation, and purification of the raw AgNPs obtained by the sonochemical method.

### Physical and chemical characterization of purified sub-15 nm AgNPs obtained by sonochemistry

#### Particle size and Particle size distribution

The purified AgNPs dispersed in the supernatant were thoroughly characterized to determine their particle size, size distribution, and shape. For the TEM analysis of the sub-15 nm AgNPs, two different functionalization strategies of the surface of the grids using PLL and Alcian blue were evaluated. Both treatments were able to generate a very thin film on the TEM grating surface that increased the hydrophilicity of the grating surface, minimizing the generation of sample deposition and drying artifacts. Also, the deposition of individual AgNPs on the surface of the grid was promoted by Van der Waals intramolecular interactions with the protonated amino groups (–NH_3_^+^) in the chemical structure of PLL and Alcian blue, mitigating the generation of agglomerations in the TEM analysis.

**Figure 4:**
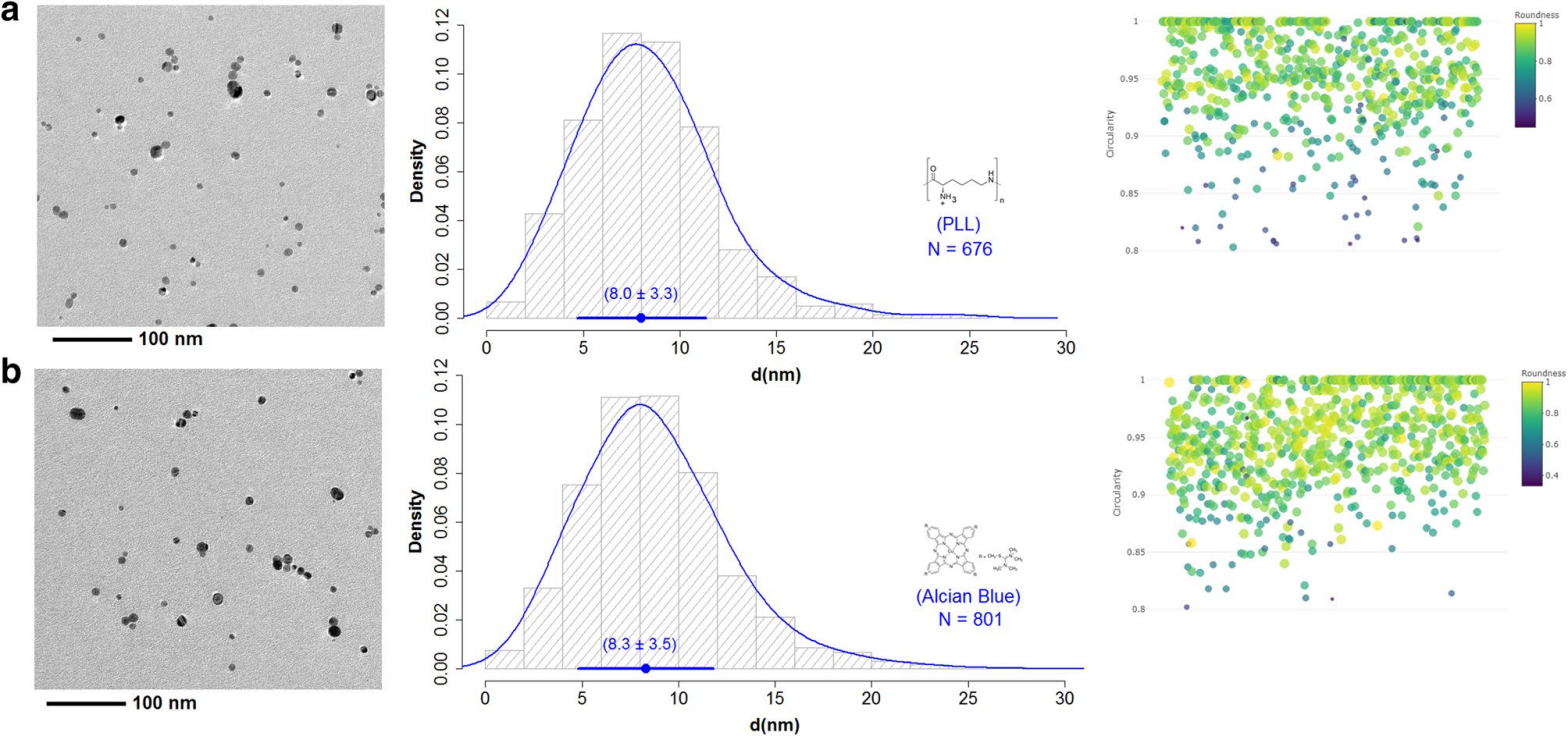
Characterization of the particle size, size distribution, and shape descriptors of the purified AgNPs by TEM. (**a**) AgNPs deposited using PLL, (**b**) AgNPs deposited using Alcian blue.

TEM size characterization of purified AgNPs was displayed in Fig. 4. TEM images showed that both deposition methods (PLL and Alcian blue) successfully promoted the deposition of clean and single NPs of the purified sub-15 nm AgNPs with a minimal contribution of agglomerates and/or artifacts. Note that more than 20 images for each deposition method were analyzed with a total number of individual particles (N) of 676 and 801, respectively. This particle number was essential to provide high statistical confidence^39,40^ and to decrease the uncertainty in the particle size determination^41^. As can be seen in Fig. 4, the particle size (median ± MAD) of AgNPs determined by TEM using PLL and Alcian blue as functionalization agents was found to be 8.0 ± 3.3 nm (Fig. 4a), and 8.3 ± 3.5 nm (Fig. 4b), respectively.

Comparison of two means (Student t-test) indicated that no significant differences at 95% confidence (p < 0.05) between both deposition methods were observed. Therefore, both deposition methods can be used indistinctly to accurately characterize the particle size of purified AgNPs. Due to the above, the final estimation of the particle size was a consensus value between both TEM deposition methods, computed by the DerSimonian–Laird model using the NIST consensus builder^42^. The estimation of the dispersion of the consensus value was carried out using a parametric bootstrap. Thus, the consensus value for particle size of the purified AgNPs was found to be 8.1 ± 2.4 nm with a 95% coverage interval that ranged from 3.4 nm to 13 nm (Supplementary Fig. S5), which statistically demonstrated that purified AgNPs obtained by sonochemical method exhibited a size distribution in the sub-15 nm scale. These results are extremely promising since despite the existence of classic wet synthesis methods capable of generating quasi-spherical and monodispersed AgNPs^1^, many of them are limited in obtaining monodispersed AgNPs with larger particle sizes, therefore this route being an interesting alternative for the direct production of sub-15 nm monodispersed AgNPs without further thermal^2^, chemical^5,10,11^, or physical^18^ treatments to improve their shape and size..

The comparison of the particle size distribution of the raw AgNPs (9.1 ± 3.3) nm, Fig. 2d the purified AgNPs (Fig. 4) demonstrated the effect of the purification step by centrifugation in the narrowing of the particle size distribution mainly derived from the efficient elimination of a large portion of AgNPs larger than 15 nm. Also, the functionalization of the TEM grids also helped to reduce the dispersion of the determination of the size distribution since it significantly minimized the formation of artifacts and/or agglomerations during TEM measurements. In the case of the TEM characterization of the raw AgNPs, the approach described above was not used in order to make a quick and practical characterization of the raw material, which was presumed to be impure and to need a subsequent purification process.

#### Particle shape and crystallinity

The particle shape was considered another relevant aspect in the characterization of the purified AgNPs. This dimensional property is important to understand the performance, functionality, as well as the possible applications of the sub-15 nm AgNPs obtain in this work^43^. Within the large number of different shape descriptors that have been suggested to characterize the shape of NPs^43^, one of the most practical approaches uses the estimation of the circularity of 2D projections from TEM micrographs^44,45^. As displayed in Fig. 4, the circularity values obtained for the purified AgNPs by TEM, were mostly distributed ($\sim$ 88%) between 0.9 and 1.0, being the value of 1.0 a perfect circle. In addition, a small portion AgNPs ($\sim$ 11%) exhibited circularity values in the range of 0.8–0.9. These results confirmed that the sub-15 nm AgNPs exhibited a high sphericity superior to other synthesis and stabilization strategies of monodispersed AgNPs that report circularities between 0.6 and 0.8^46^. The high roundness, another typical shape descriptor, observed for the sub-15 nm AgNPs with values ranging from 0.8 to 1.0, demonstrated that the purified AgNPs exhibited a high degree of curvature at the surface, which confirmed the presence of mostly quasi-spherical shapes with some low irregularities on the surface.

**Figure 5:**
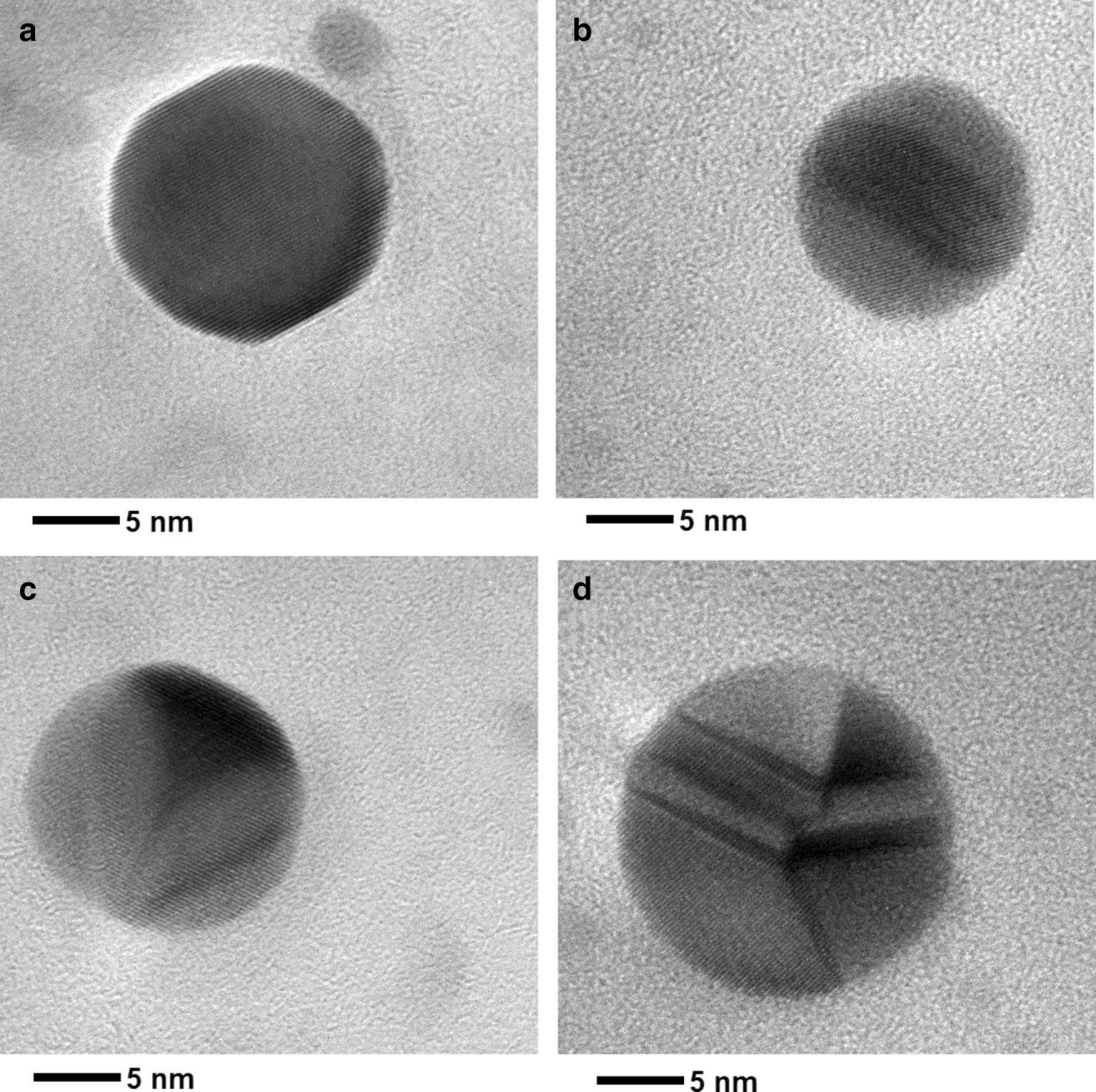
HR-TEM micrographs of the sub-15 nm AgNPs obtained by sonochemistry. (**a**) AgNPs with near-spherical shape, (**b**,**c**) twin-fault AgNPs with near-spherical shape, (**d**) twin-fault AgNPs containing parallel-twin lamellae.

HR-TEM analysis (Fig. 5a,b) confirmed all the previous finding reported by TEM for the purified AgNPs, particularly the quasi-spherical shape. Also, Fig. 5c,d showed AgNPs with highly spherical shapes, but with variations in their crystalline structure that could generate a loss of roundness at the surface level of the NPs, which was in good agreement with the results previously described by TEM. Mainly, from a detailed exploration of the HR-TEM images (Fig. 5) it can be observed that the purified AgNPs were mostly single-crystal particles of quasi-spherical shape (Fig. 5a). However, other crystalline structures like single twin-fault particles of quasi-spherical shape (Fig. 5b,c) and twin-fault particles containing parallel-twin lamellae (Fig. 5d) were also evidenced. The existence of these types of nanocrystals may be primarily due to the growth conditions during the first minutes of the sonochemical synthesis. In this context, it is well known^45^ that ultrasonic cavitation generates hot spots with temperatures of ~ 5000 °C, pressures of approximately 1000 atm and heating and cooling rates in excess of 10^10^ K s^−1^, which facilitates the generation of dramatic changes of the crystal structures of the materials. In fact, the standardization of the ultrasonic power of the sonochemical system (Supplementary Fig. S6, blue dots) shows higher temperature gradients ($\Delta T\sim 8.4 ^\circ \mathrm{C})$ from 0 to 8 min of reaction. Then, temperature stabilizes ($\Delta T\sim 0.1 ^\circ \mathrm{C})$ and remains almost invariable in the final stage (16 min) of the ultrasound irradiation. Also, as can be seen in Supplementary Fig. S6 (orange dots), the energy reached 1601 J at the end of the ultrasound irradiation (16 min), that is equivalent to an acoustic energy density of 0.040 W mL^−1^ which is enough power per unit of volume to obtain 50 mL of raw AgNPs. The previous results are consistent with that reported by other sonochemical syntheses of metallic NPs^47^, demonstrating that this new approach have enough acoustic energy density to promote the generation of small particles, modifying the crystal structures of the AgNPs as well as to generate secondary products (aggregation/agglomerations) as described above. In addition, it is important to highlight sonochemical synthesis typically promotes the coalescence or fusion of NPs as a mechanism of growth^35^; therefore, the changes in the crystalline structure of the NPs are primarily due to the ultrasonic treatment performed during the first minutes (8 min) of AgNPs synthesis.

#### Elemental composition analysis

**Figure 6:**
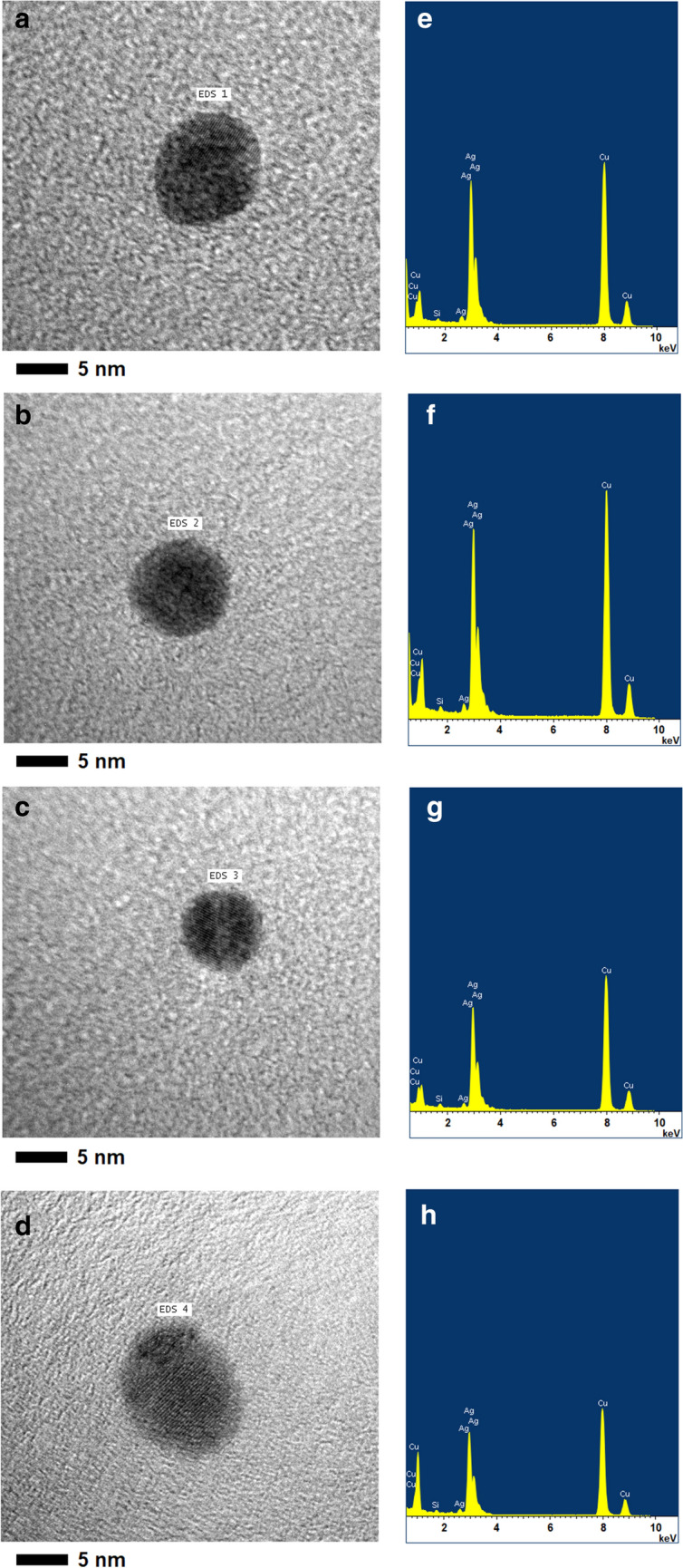
TEM-EDS analyses of the sub-15 nm AgNPs obtained by sonochemistry (**a**–**d**) HR-TEM measurements, (**e**–**h**) EDS spectra.

Figure 6a–d shows four independent TEM-EDS analyses performed to the purified AgNPs with sizes ranging from ~ 8 to ~ 11 nm. The EDS spectra obtained in all measurements, (Fig. 6e–h), show five peaks located between 2 and 4 keV that can be completely associated with the silver characteristic lines K and L, demonstrating that AgNPs core is constituted by only Ag atoms. In addition, the spectra do not show peaks attributable to the presence of oxygen (O), suggesting that the purified AgNPs did not experiment any oxidation processes during the synthesis and further storage process. This is in agreement with the stability results that will be shown later, where the high stability of the purified AgNPs can also be associated with the absence of Ag_2_O on their surface or constitution, decreasing the probability of generating destabilization processes of the colloidal suspension (e.g., agglomerations and/or aggregations) or increasing its solubility in aqueous media due to the present of Ag_2_O (K_ps_ of Ag_2_O ~ 1.52·10^−8^ at 20 °C). On the other hand, EDS spectra show peaks attributable to the presence of Cu and Si, which are characteristic and completely attributable to the TEM grid used for the deposition and analysis of the samples by TEM-EDS.

#### *Optical properties and bandgap energy (E*_*g*_*)*

Supplementary Fig. S7 shows the comparison of UV–Vis spectra for crude AgNPs and purified AgNPs. As can be seen, the most noticeable change is the decrease in Abs_MAX_ ($\sim$ 51%), that indicates a significant decrease in the particle concentration of AgNPs in suspension as a result of the purification process by centrifugation. Only slight changes in the FWHM ($\Delta$ FWHM = 0.04 nm) and λ_SPR_ ($\Delta {\lambda }_{SPR}$= 4 nm) were observed that may be attributable to the elimination of aggregates/agglomerates during the purification process that improves the definition and symmetry of the la SPR band.

Supplementary Fig. S8a–c shows the UV–Vis characterization of three independent samples (S_1_, S_2,_ and S_3_) of purified AgNPs. The average value of the optical properties generated by the SPR band were 402 nm, 65.94 nm, and 1.0874 for the λ_SPR_, FWHM, and Abs_MAX,_ respectively. From the results, the λ_SPR_ confirmed the presence of AgNPs with small particle size, and the low FWHM suggested that the purified AgNPs had relative low polydispersity of the particle size distribution, both results agree with the obtained by TEM and HR-TEM. Also, the Abs_MAX_ in all the samples revealed a high concentration of NPs in the purified suspension despite being subjected to a purification process by centrifugation. Finally, E_g_ of the purified AgNPs can be determined indirectly by absorption spectra following Tauc’s equation^48^. Supplementary Fig. S8d–f shows the E_g_ obtained for three independent samples of purified AgNPs. An average Eg of 2.795 eV with an expanded uncertainty (k = 2) of ± 0.002 eV was obtained, which is very similar with the results reported by AgNPs with particle size smaller than 10 nm synthesized by high temperature ($\sim$ 400 °C) thermal treatment method^49^. More details about the determination of E_g_ and the computation of its associated uncertainty can be found in the supporting information.

### Evaluation of the stability of the sub-15 nm AgNPs under different conditions

#### Stability of sub-15 nm AgNPs under UV radiation (short-term stability)

Stability in terms of NPs size is defined as the preservation of the dimensionality of the NPs during storage and/or experiment^50^. The limiting factor that hampers the implementation of sub-15 nm AgNPs with well-defined properties in the analytical and bioanalytical fields is the limited stability because the high tendency to agglomeration, aggregation, and/or dissolution processes. For this reason, a thorough evaluation of the stability of the synthesized sub-15 nm AgNPs under different controlled storage and exposure conditions required to realistically assess their potential applications and lifetime. In this context, monitoring the optical properties of purified AgNP suspensions under continuous exposure to UV radiation was selected as an accelerated test to evaluate the impact of a highly energetic stimulus on the most relevant properties (median particle size, size dispersion, and concentration) of AgNPs.

**Figure 7:**
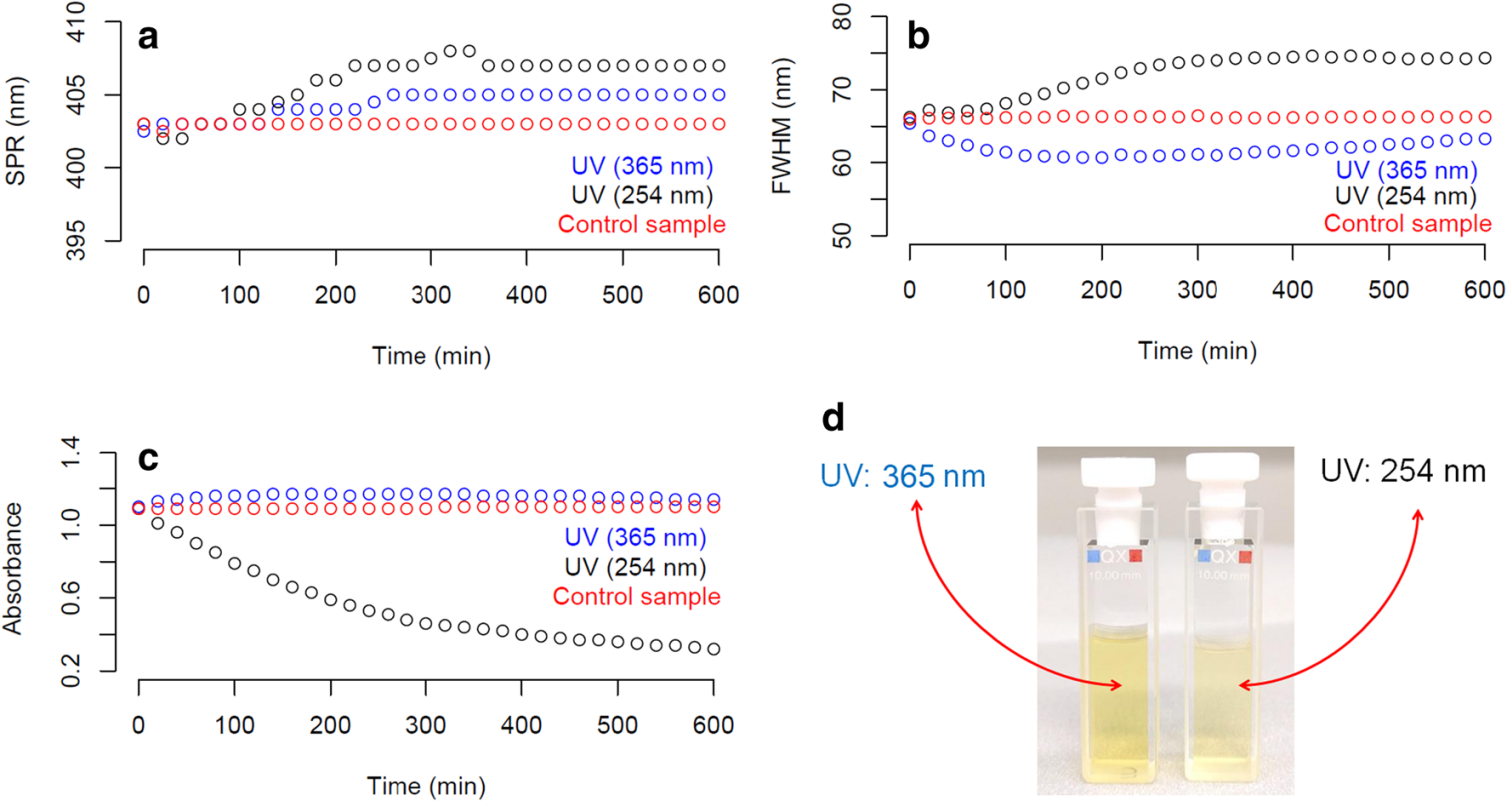
Short-term stability of sub-15 nm AgNPs exposed to UVC (254 nm) and UVA (365 nm) radiation. (**a**) λ_SPR_, (**b**) FWHM, (**c**) Abs_MAX_, (**d**) AgNP suspensions after UVC and UVA exposition. Black dots ($^\circ$) represent measurements of AgNPs exposed to UVC radiation, blue dots ($^\circ$) represent measurements of AgNPs exposed to UVA radiation, red dots ($^\circ$) represent measurement of the control sample.

The evolution of the optical properties (SPR, Abs_MAX_, and FWHM) of the sub-15 nm AgNPs when they were exposed to two types of irradiations in the UV region of the spectrum, UVA (365 nm) and UVC (254 nm), was monitored every 20 min over a period of 600 min as illustrated in Fig. 7. In this study, a control sample was stored in the dark, covered with aluminum foil, and kept under laboratory room conditions (20 °C, and relative humidity of 60%).

As can be seen in Fig. 7 (blue dots) irradiation of the sub-15 nm AgNPs with UVA radiation induced a change in the optical properties of the AgNPs from the beginning of the irradiation. In Fig. 7a it can be observed how λ_SPR_ (401 nm) was gradually shifted towards higher wavelengths until reaching a plateau at 405 nm for 300 min of irradiation. This shift in λ_SPR_ correlated with an increase in the particle size, as described elsewhere^14^. Exposure to UVA radiation also caused a small increase in Abs_max_, which corresponded with a small increase of the concentration of AgNPs in suspension. These results suggest that UVA radiation has the ability to photo-induce an increase in the size of the sub-15 nm AgNPs, as well as to generate the formation of new individual AgNPs due to its ability to oxidize the Cit^-^ of the surface of AgNPs^51^. The trend in λ_SPR_ also correlated with the results observed for FWHM (Fig. 7b), that gradually decreased reaching a minimum at 200 min of UVA exposure. Subsequently, the FWHM increased slightly, however, it remains below that observed in the control sample (Fig. 7b, red dots). Therefore, the above result indicates that UVA radiation significantly reduced the polydispersity of the size distribution of sub-15 nm AgNPs over time, which opens the opportunity for development of new routes or treatments for the production of small AgNPs without changing significantly the particle size, particle concentration, and improving its polydispersity of the size distribution.

A different behavior was observed for the stability study of AgNPs when under UVC (254 nm) irradiation. While both λ_SPR_ and FWHM gradually increased over time, Abs_MAX_ drastically decayed as a function of the exposure time to UVC radiation (Fig. 7c). The decrease of Abs_MAX_ by 30%, and in turn of the concentration of sub-15 nm AgNPs, clearly evidenced the destabilization of the metallic cores likely caused by a photo-oxidative mechanic associated with the high energy of the UVC radiation. As visual confirmation of the decrease in the concentration of AgNPs as a result of the exposure to UVC radiation, Fig. 7d showed a significant change of the intensity of the yellow coloration of the original batch to an almost translucent yellow. While similar result has been previously reported for AgNPs with average particle sizes ranging from 20 to 80 nm^52^, to the best of our knowledge this is the first time that the short-term stability of sub-15 nm AgNPs under UVA and UVC radiation has been assessed.

Finally, it is important to highlight that when the sub-15 nm AgNPs were exposed to UVA radiation for a period longer than 24 h (data not shown), the initial yellow hue of the AgNP suspension was transformed towards a near colorless solution due to the loss of the SPR absorbance, which indicates a complete photo-oxidation of the metal nuclei and transformation of the AgNPs to Ag^+^_(aq)_ ionic. On the other hand, the optical properties of the control sample of AgNPs (Fig. 7, red dots) remained highly stable during the experiment, which demonstrates that the sub-15 nm AgNPs produced by the sonochemical method exhibited a great short-term stability (600 min) when stored in the dark and under laboratory room conditions (20 °C, and relative humidity of 60%).

### Stability of sub-15 nm AgNPs at different storage temperatures (long-term stability)

The assessment of the long-term stability of the sub-15 nm AgNPs at two different storage temperatures (4 °C and 20 °C) was carried out through the monitoring of the evolution of the optical properties over a period of 24 weeks adopting a practical approach based on classical stability studies of reference materials^53^. If the change of the optical property is small, it is possible to determine an ordinary least squares (OLS) model that empirically relates the optical property and storage time. If the slope ($\widehat{\beta }$) of the OLS model does not vary significantly from zero ($\widehat{\beta }$ = 0) at a 95% confidence level it can be established with some degree of confidence that there is no appreciable change in the stability of λ_SPR_, FWHM, and Abs_MAX_ of the nanoparticulate suspension and, therefore, in the stability of particle size, size distribution, and concentration of AgNPs properties of the sub-15 nm AgNPs, respectively. Hence, the described approach becomes a very practical tool to study the long-term stability of metallic NPs.

**Figure 8:**
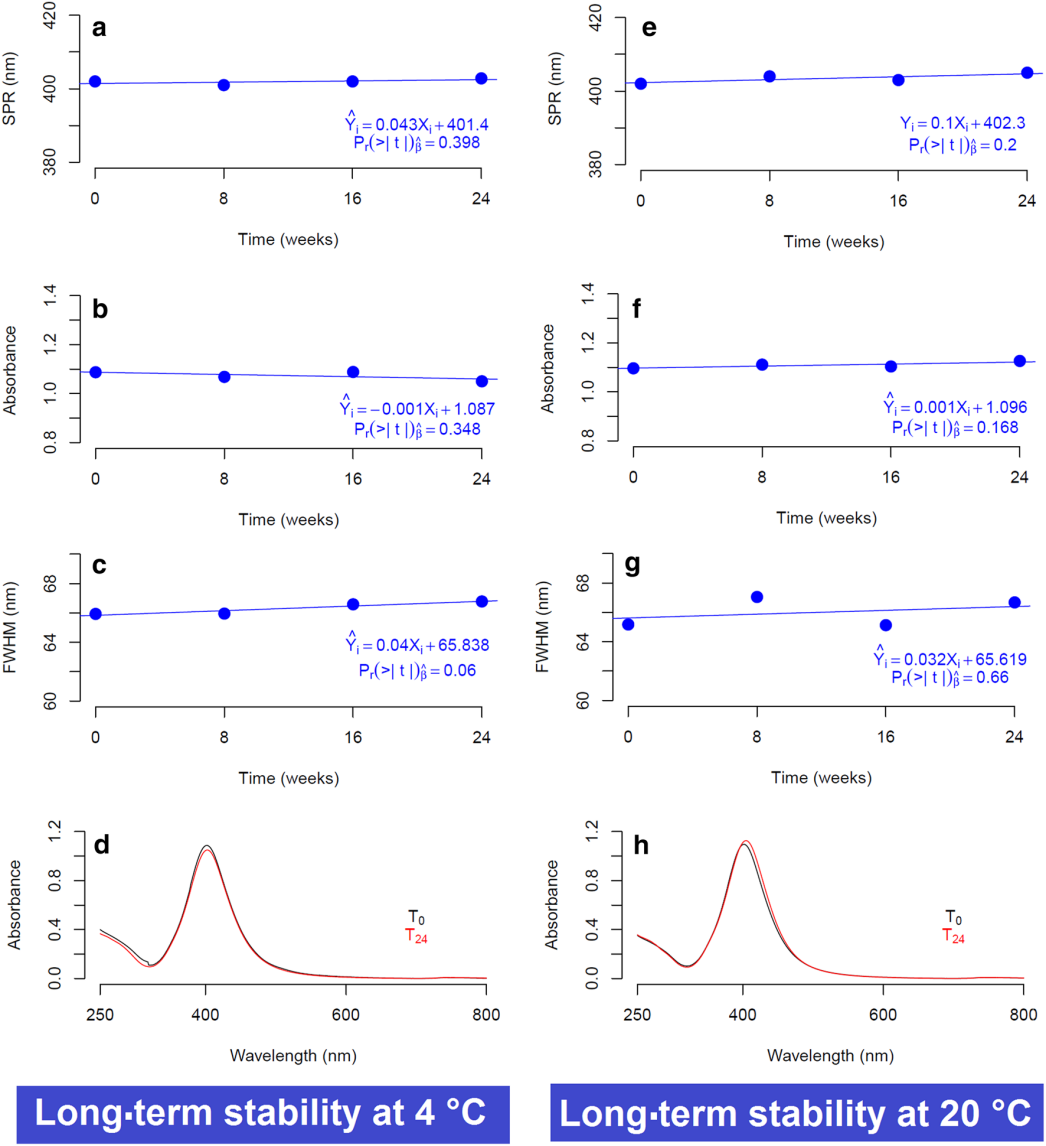
Long-term stability of the sub-15 nm AgNPs stored at different temperature conditions (4 °C and 20 °C). The images illustrate the OLS models of each optical property of the sub-15 AgNPs. The model OLS and t-Student test performed to the slope ($\widehat{\beta }$) is present in all the images.

OLS regressions for each of the optical properties (λ_SPR_, Abs_MAX_, FWHM) of the sub-15 nm AgNPs obtained by the sonochemical method were plotted in Fig. 8 for both storage conditions (4 °C and 20 °C). In general, the slopes of the regressions did not vary significantly from zero ($\widehat{\beta }$ = 0) with a level of confidence of 95% using a t-Student test with two degrees of freedom. Remarkably, for the storage at 4 °C (Fig. 8a), λ_SPR_ remained practically unchanged (∆SPR ~ 1 nm) after 24 weeks, with a slope statically equal to zero (${\widehat{\beta }}_{4^\circ \mathrm{C}}^{SPR}$ = 0.043 nm weeks^−1^), which proved that the sub-15 nm AgNPs did not experience a significant change in the median particle size. Furthermore, Abs_MAX_ decreased by less than 2% during 6 months of storage, resulting in a negative slope (${\widehat{\beta }}_{4^\circ \mathrm{C}}^{Abs}$ = − 0.001 weeks^−1^) that indicated a minimal reduction of AgNPs concentration over time (Fig. 8b). It looks pretty likely that the slight loss in the absorbance was caused by the formation of a small portion of agglomerates during the long storage period; a process kinetically unfavorable due to the low Brownian motion of metallic NPs^54^ at low temperatures. In any case, the t-Student test indicated that ${\widehat{\beta }}_{4^\circ \mathrm{C}}^{Abs}$ did not differ significantly from $\widehat{\beta }$ = 0 with a level of confidence of 95%, so it was possible to conclude that the concentration of AgNPs did not vary significantly during the 6 months of storage. Similarly, while ${\widehat{\beta }}_{4^\circ \mathrm{C}}^{FWHM}$ did not significantly differ from $\widehat{\beta }$ = 0, the slight increase in FWHM observed in Fig. 8c might also be attributed to the presence of a tiny portion of agglomerates as discussed above. The high long-term stability of the optical properties of sub-15 nm AgNPs at 4 °C was illustrated with the perfect overlap of UV–Vis spectrum acquired at the beginning (T_0_) and at the end of the stability study at 24 weeks (T_24_) as can be seen in Fig. 8d.

On the other hand, a high long-term stability of the optical properties of the sub-15 nm AgNPs stored at 20 °C was observed, similar to that previously described at 4 °C. Surprisingly, while ${\widehat{\beta }}_{20^\circ \mathrm{C}}^{SPR}$ did not significantly differ from $\widehat{\beta }$=0, ${\widehat{\beta }}_{20^\circ \mathrm{C}}^{SPR}$ was found to be approximately the double larger than ${\widehat{\beta }}_{4^\circ \mathrm{C}}^{SPR}$ (Fig. 8e). Thus, long term storage at 20 °C seemed to cause a more significant shift of λ_SPR_ (~ 2 nm) and consequently of the particle size than 4 °C. This finding agreed with the theories of NPs growth that described that a rise in temperature can boost the diffusion of leftover monomers (precursor) around the surface of NPs^34^. In the case of the dispersion of the size distribution (Fig. 8f) and concentration (Fig. 8g) of the sub-15 nm AgNPs, the stability study showed a slight increase of FWHM, Abs_MAX_ (~ 2%), as well as a higher variability of the OLS model residuals for the storage at 20 °C. These results could be attributed to Ostwald ripening processes between the thermodynamically less stable AgNPs^34^. However, further studies with alternative analytical techniques such as small-angle X-ray scattering (USAXS) or in situ liquid TEM would be necessary for a more definite identification of the mechanisms responsible for the change in the optical properties during long-term storage. The comparison between UV–Vis spectra of AgNPs at the beginning of the stability study (T_0_) and at the end of the stability study at 20 °C for a period of 24 weeks (T_24_), confirmed a minimal change in the shape of the spectra UV–Vis, as observed for storage at 4 °C (Fig. 8h).

Overall, the high long-term stability achieved for the synthesized sub-15 nm AgNPs is a very promising finding compared to the stability results reported for AgNPs with similar stabilizing agents and particle size. Specifically, a stability study was conducted for ~ 6 nm citrated-capped AgNPs, synthesized by chemical reduction method, stored at laboratory temperature over a period of 6 months^55^. A noticeable change in the UV–Vis spectrum, with a substantial decrease (~ 30% loss) of Abs_MAX_, as well as a redshift of λ_SPR_ were reported, which suggests a decrease in AgNP concentration and an appreciable increase in particle size. The difference in long-term stability between both studies could be attributed to the novel pathway proposed in this study.

## Conclusions

The present investigation demonstrates the potential of a new sonochemical synthesis route to produce sub-15 nm AgNPs with high sphericity and notorious high stability in aqueous media in comparison with other classical synthesis routes. A deeper understanding of the evolution of nucleation, growth, and destabilization processes of AgNPs during sonochemical synthesis was achieved through multi-technique characterization. Alternatively, a simple and direct purification strategy based on centrifugation allowed an efficient removal of larger secondary products from raw AgNPs. Different functionalization strategies for the deposition of purified AgNP suspensions consistently revealed the existence of a median particle in the sub-15 nm scale by using TEM. The outstanding imaging resolution of HR-TEM confirmed the quasi-spherical nature of the AgNPs with a high level of circularity and roundness. Furthermore, the existence of different types of twin-faults in the crystalline structure of AgNPs was attributed to the high energy of the sonochemical treatment.

The analysis of the optical properties under UV light exposure revealed that the AgNPs synthesized by the sonochemical method exhibited high short-term stability under laboratory conditions. While UVC radiation significantly impacted the stability of the AgNPs metal nuclei, increasing the particle size, generating a greater polydispersity, and decreasing substantially the particle concentration, UVA radiation caused improvement on some optical properties and consequently of their dimensional properties. This finding opens the door to the development of new routes or treatments for the production of small AgNPs with high stability and improved dimensional properties.

It is expected that the good long-term stability, achieved for the optical properties of the synthesized sub-15 nm AgNPs over a period of six months under different storage conditions (4 °C and 20 °C), can extend the applicability of sonochemical synthesis to the development of advanced applications in varying fields such as the production of new NP reference materials, nanometrology, nanocatalysis, photovoltaic cells, nano-fluidics, innovative textile applications, medicine, biomedicine, and biosensors, among others.

## Methods

### Chemicals

Chemicals used during this study were used as received without further purification. High-purity water (≥ 18 MΩ cm at 25 °C resistivity, Millipore, MA, USA) was used to prepare all solutions involve in the sonochemical synthesis. Silver nitrate (AgNO_3_), 99,999% (w/w), sodium borohydride (NaBH_4_) 99% (w/w), trisodium citrate dehydrate (NaCit), poly-l-lysine (PLL) 0.1% (w/v) in H_2_O and Alcian blue 8GX in powder were purchase form Sigma Aldrich (MO, USA). Ethanol 95% (v/v) was purchased from Merck (MO, USA). Carbon/Formvar film coated copper grids (400 mesh) were acquired from Ted Pella Inc. (CA, USA) were used for TEM and HR-TEM. Freshly cleaved mica surfaces were used as substrate for AFM characterization. Nitrogen (N_2_) with ultra-high purity (UHP $\sim$ 99%) was purchased form Praxair Technology (AL, USA). Standard Reference Material^®^ 2034^56^ was used as transfer standard for the verification, drift control, and calibration of the wavelength scale of UV–Vis absorption spectrophotometers. All glassware was washed with HNO_3_ 10% (w/v), rinsed with high-purity water, and oven dried before use.

### Sonochemical reaction system

The system consisted of a 250 mL round bottom flask with three 14/20 necks (Sonics Materials, CT, USA) coupled using an adapter (Sonics Materials, CT, USA) to a 700 W power sonicator with an adjustable pulse (QSonica, CT, USA). A solid titanium sonicator probe with a diameter of 12.7 mm was inserted inside the main neck. 40 mL of NaBH_4_ 2.0 mM were placed inside the sonochemical reaction vessel. The precursor agent (AgNO_3_, 1.0 mM) was continuously introduced at 0.60 g min^−1^ using a capillary tube until the NaBH_4_ solution reached a temperature of (0.0 ± 0.4) °C, that corresponded to a period of 30 min of thermostatic equilibrium in a thermoregulated bath. To maintain a constant nitrogen atmosphere inside the flask to prevent any oxidation of the AgNPs surface during the synthesis, a needle with nitrogen flow was used. This flow was set to 1 mL min^−1^ by means of a rotameter (Cole-Palmer, IL, USA). The third neck was used as a purge of the N_2_ and prevention of overpressure in the reaction system using an adapter with double entry. The control of the reaction temperature at 0 °C was carried out by immersing the sonochemical reaction vessel in a Lauda Ecoline RE104 bath (LK, Germany). Standardization of ultrasonic power for the sonochemical reaction system was performed following the calorimetry approach detailed in Ref.^57^.

### UV–Vis measurements

#### UV–Vis on-line monitoring synthesis system

An on-line UV–Vis system, similar to the reported elsewhere^58^, was used to monitor and study the evolution of the optical properties during the sonochemical synthesis reaction of the raw AgNPs (Supplementary Fig. S1a). For this purpose, the sonochemical reaction system described above was coupled with a peristaltic pump to introduce and recirculate the sample into the UV–Vis spectrophotometer (Lambda 950, PerkinElmer, MA, USA) using a quartz flow cell (Helma^®^ absorption cell, Mülheim, Germany) with a path length of 2 mm and a chamber volume of 124 μL.

#### UV–Vis measurements

Optical properties of the purified AgNPs were determined as described elsewhere^58^ using the same instrumental conditions and spectrophotometer described in the previous section. For this instance, three subsamples of the purified AgNPs were transferred into 10 mm × 10 mm quartz cells transparent to UV radiation to perform this UV–Vis measurements. Eg of the purified AgNPs was determined by measuring three independent UV–Vis spectra. Supplementary Table S2 described the equations involved in the determination of Eg using Tauc’s equation^48^. Also, description about the estimation of the mean Eg (Supplementary Table S3) and measurement uncertainty is described in Supplementary Table S4.

### Atomic force microscopy (AFM)

AFM measurements for the monitoring of the sonochemical synthesis were performed following the ASTM E2859-11^59^ Standard Guide. Briefly, 25 µL of undiluted raw AgNPs were deposited on a cleaved mica substrate previously activated with a 0.1% PLL solution that provides a positively charged surface able to bond the AgNPs. Once AgNPs were bonded and air-dried on the mica, images were captured with an Asylum Research model MFP-3D SA (Oxford Instruments, Abingdon, UK) in non-contact (tapping) mode using a cantilever with a radii tip < 10 nm and stiffness of 48 N m^−1^. Scan sizes of 3.0 µm × 3.0 µm with 512 lines and a scan speed of 1 Hz (per scan line) were used to image. Image visualization and particle height analysis were performed using Gwyddion (version 2.57) modular program^60^.

### Transmission electron microscopy (TEM), high-resolution transmission electron microscopy (HR-TEM), and energy dispersive X-rays spectroscopy (EDS)-TEM

The "*grid on drop*" method was used as a quick and practical approach to deposit and characterize the raw AgNP suspensions coming from sonochemical synthesis. Specifically, 5 µL of undiluted AgNPs were deposited on the TEM grids and then dried for at least 24 h. In the case of the nano-characterization of purified AgNPs, TEM grids (400 mesh Cu-Carbon/Formvar film-coated) were previously treated using two different deposition methods: Alcian Blue 1% (w/v) solution based on the protocol proposed by Mast Demeestere^61^, and PLL 0.1% (w/v) solution recommended elsewhere^62^. TEM measurements were performed on a JEM 2011 (JEOL, Tokyo, Japan) instrument, in bright-field mode operating at 120 kV and using magnifications of approximately between 40,000 and 80,000×. The micrographs (1024 pixels × 1024 pixels in size) were recorded using a 1 k × 1 k CCD camera (794, Gatan). HR-TEM images of the purified AgNPs were recorded using the same instrument using a magnification of approximately 600,000×. Finally, TEM-EDS analysis was performed using a 6498 EDS detector (Oxford Instrument, United Kingdom) coupled to the TEM instrument previously described.

### Centrifugation and purification

The purification of the raw AgNPs was performed using a Sorvall WX Ultra Series, WX 80 Ultracentrifuge (Thermo Fisher Scientific, MA, USA) with a Beckman SW 50.1 swinging bucket rotor (max. 5 mL) where 3.0 g of the NPs suspension were placed. Samples were vacuum centrifuged using a relative centrifugal field (RCF) of 10,000*g*, 20,000*g*, and 30,000*g* for 20 min with a deceleration time of 5 min at a temperature of 4 °C. Finally, the supernatant from the centrifugation was carefully separated from the pellet deposited at the bottom of the test tubes. Both centrifugation products (supernatant and pellet) were analyzed by TEM, as detailed above.

### Purified AgNPs subsamples

A 25 mL batch of previously purified AgNPs was homogenized in an ultrasonic bath for 5 min to avoid possible agglomerations. The batch was subsequently divided into 1 mL subsamples (total of 25 subsamples). These subsamples were stored in 2 mL glass vials and in an inert N_2_ (UHP) atmosphere. The vials were covered with aluminum foil and stored in the dark to prevent any photo-degradative effect during the stability studies. A total of 5 subsamples were randomly taken to perform the UV irradiation stability study (short-term stability). Then, 16 subsamples (Supplementary Fig. S9) were randomly taken to perform the long-term stability study. Specifically, 8 subsamples were selected and placed in a refrigeration system (True^®^, USA) at (4 ± 2) °C, while the remaining 8 samples were placed in an incubator (Revco, USA) at (20 ± 1) °C. The temperature control of the isothermal medium was carried out with previously calibrated K-type thermometers (Fluke T300 FC). Finally, 3 subsamples were selected to perform the UV–Vis measurements of the purified AgNPs.

### Stability of the sub-15 nm AgNPs

#### Short-term stability (UV irradiation)

A total of 5 subsamples were randomly taken from the purified AgNPs batch (Supplementary Fig. S2) to perform the UV irradiation stability study. For this instance, AgNPs subsamples were transferred into 10 mm × 10 mm quartz cells transparent to UV radiation. A Spectroline CX-20 UV camera was used to irradiate the sub-samples with both UV radiations. Two subsamples were used to study the effect of short-wavelength UV (UVC) radiation (λ: 254 nm, and intensity of 500 μW/cm^2^) on AgNPs. Other two subsamples were used to study the effect of the relative long-wavelength UV (UVA) radiation (λ: 365 nm, and intensity of 610 μW/cm^2^) on AgNPs. In both studies, subsamples were irradiated over a period of 20 min before performing absorbance measurements, having a total cumulative exposure of 600 min. Finally, one sub-sample was used as a control, which was kept in the dark, covered with aluminum foil and stored at room temperature (20 °C). The optical properties of the AgNPs were determined using the spectrophotometric scanning conditions and processing capabilities described in the UV–Vis characterization section.

#### Long-term stability (different storage temperatures

Two reference temperatures (4 ± 2) °C and (20 ± 1) °C were selected for the evaluation of the long-term storage stability over a period of 6 months. As described previously, eight samples randomly chosen were stored at each temperature. UV–Vis absorbance of two random subsamples was measured at 0, 2, 4, and 6 months. The optical properties of the AgNPs were determined using the measurement protocol described in the UV–Vis measurements section.

### Data processing

All the calculations, statistical and graphical analyses were performed using the statistical software R. RStudio was used as an integrated development environment for the developing the programming codes. Statistical packages such as Plotly^®^^63^ and MASS^64^ were used to develop various graphs and statistical analysis in this research. As described above, *NanoUV-Vis* app^58^ was used to process and visualize data from UV–Vis spectrometric measurements of AgNPs. Finally, *NIST Consensus Builder* web application^42^ was used to estimate the consensus value of the particle size and size dispersion of the sub-15 nm AgNPs.

### Ethics declarations

The authors declare no human or animal subjects were included and no informed consent was needed in this research.

## Supplementary Information

Supplementary Information.

## Data Availability

The datasets analyzed during the current study are available from the corresponding author on reasonable request.
