# Supplementary material for: Novel pathway for the sonochemical synthesis of silver nanoparticles with near-spherical shape and high stability in aqueous media
Source: Sci Rep. 2022 Jan 18;12:882. doi: 10.1038/s41598-022-04921-9 (PMC8766478; doi:10.1038/s41598-022-04921-9)
Supplement: Supplementary file 1 — Supplementary Information. [file 41598_2022_4921_MOESM1_ESM.docx]

**Supporting Information**

**Novel pathway for the sonochemical synthesis of silver nanoparticles with near-spherical shape and high stability in aqueous media**

**Bryan Calderón-Jiménez^1,2,3*^, Antonio R. Montoro Bustos^4^, Reinaldo Pereira Reyes^2^, Sergio A. Paniagua^2^ and José R. Vega-Baudrit^2^**

^1^Chemical Metrology Division, National Metrology Laboratory of Costa Rica (LCM), 11501-2060, Costa Rica.

^2^National Laboratory of Nanotechnology, National Center of High Technology, 1174-1200, Costa Rica.

^3^Ph.D Program in Natural Science for Development (DOCINADE), Technological Institute of Costa Rica, National University, State Distance University, 159-7050, Costa Rica.

^4^Material Measurement Laboratory, Chemical Sciences Division, National Institute of Standards and Technology, Gaithersburg, Maryland, 20899, United States of America.

Correspondence to: [bcalderon@lcm.go.cr](mailto:bcalderon@lcm.go.cr)


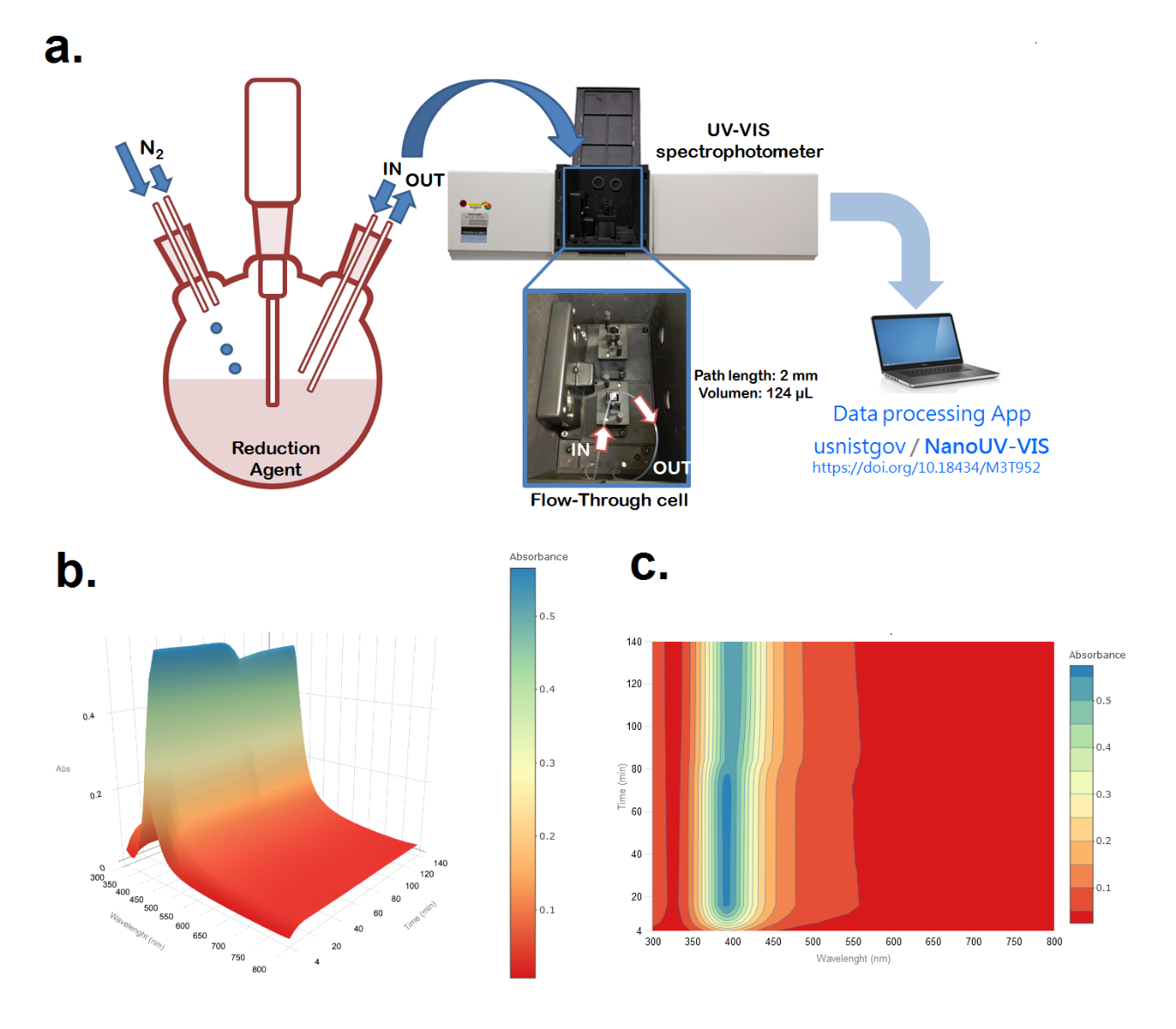


**Figure S1.** Visualization of the evolution of the AgNPs **(a)** 3D surface plot of the AgNPs optical properties during the synthesis reaction. **(b)** 2D contour plot of the AgNPs optical properties during the synthesis reaction **(c)** illustration of the on-line UV-Vis system to monitor and study the evolution of the optical properties.


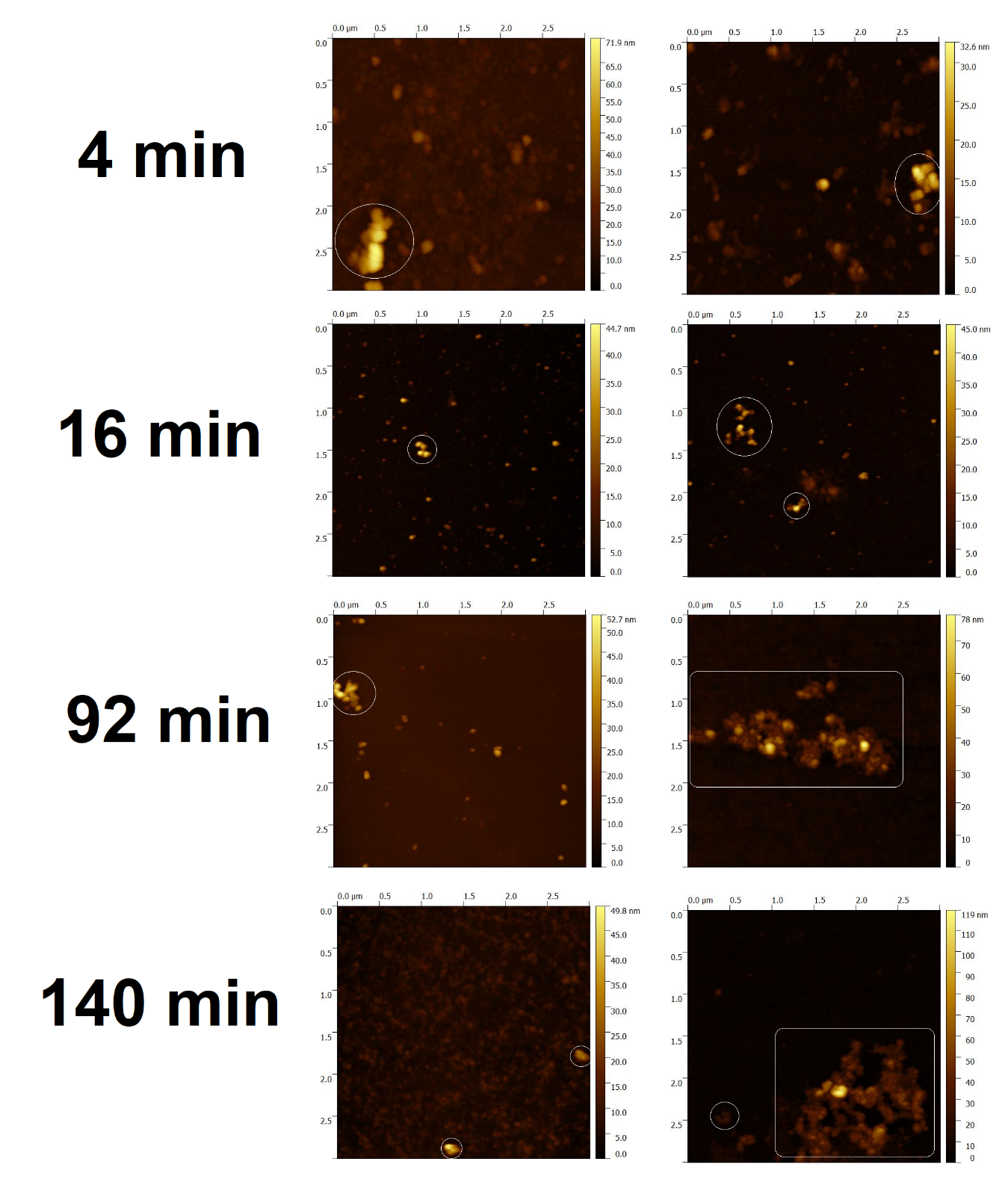


**Figure S2.** AFM images of two independent samples measured during the monitoring of the synthesis evolution to identify the generation of aggregation and agglomerations.


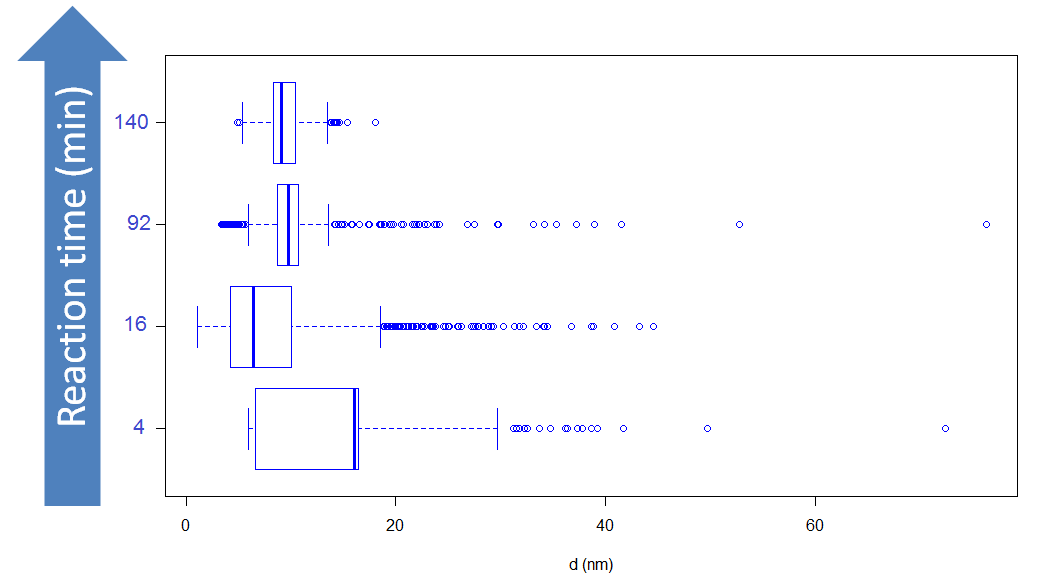


**Figure S3.** Particle size distribution obtained by AFM during the evolution of the synthesis reaction of AgNPs by sonochemistry.





**Figure S4.** TEM images of aggregations promoted by the ultrasonic treatment.


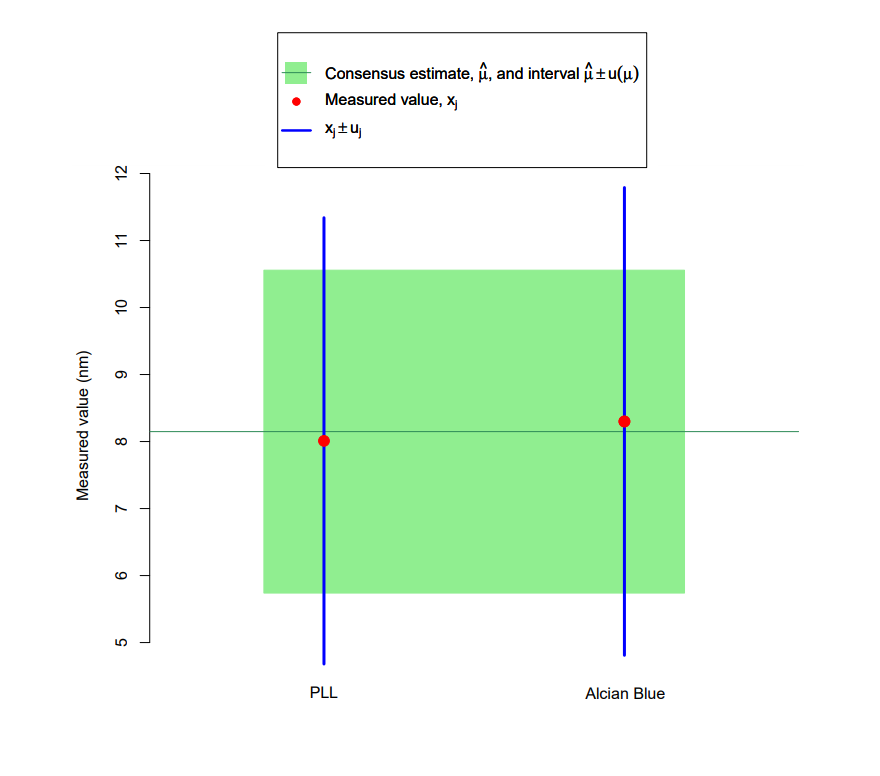


**Figure S5.** Consensus value for particle size and coverage interval of the synthesized sub-15 nm AgNPs.


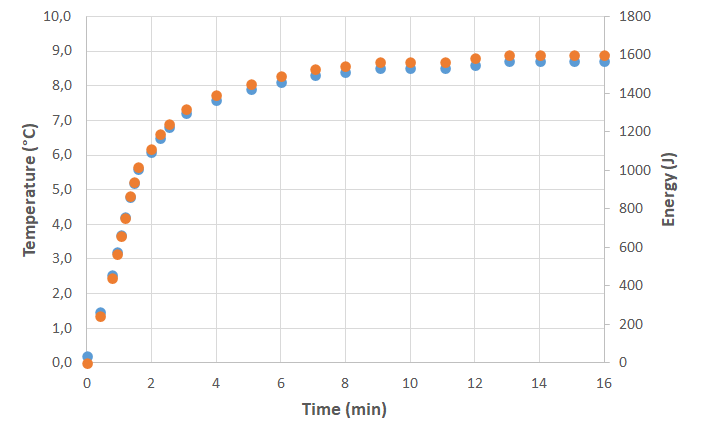


**Figure S6.** Standardization of ultrasonic power for the sonochemical synthesis of AgNPs.


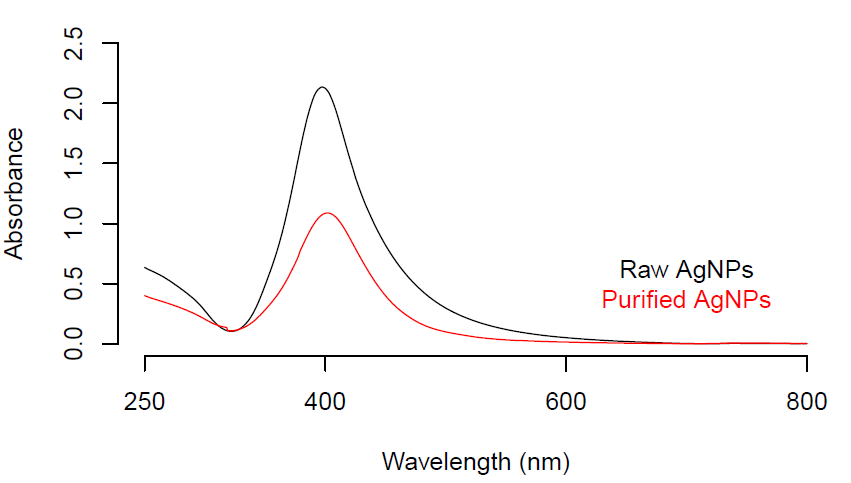


**Figure S7.** UV-Vis spectrum comparison between raw AgNPs and Purified AgNPs.


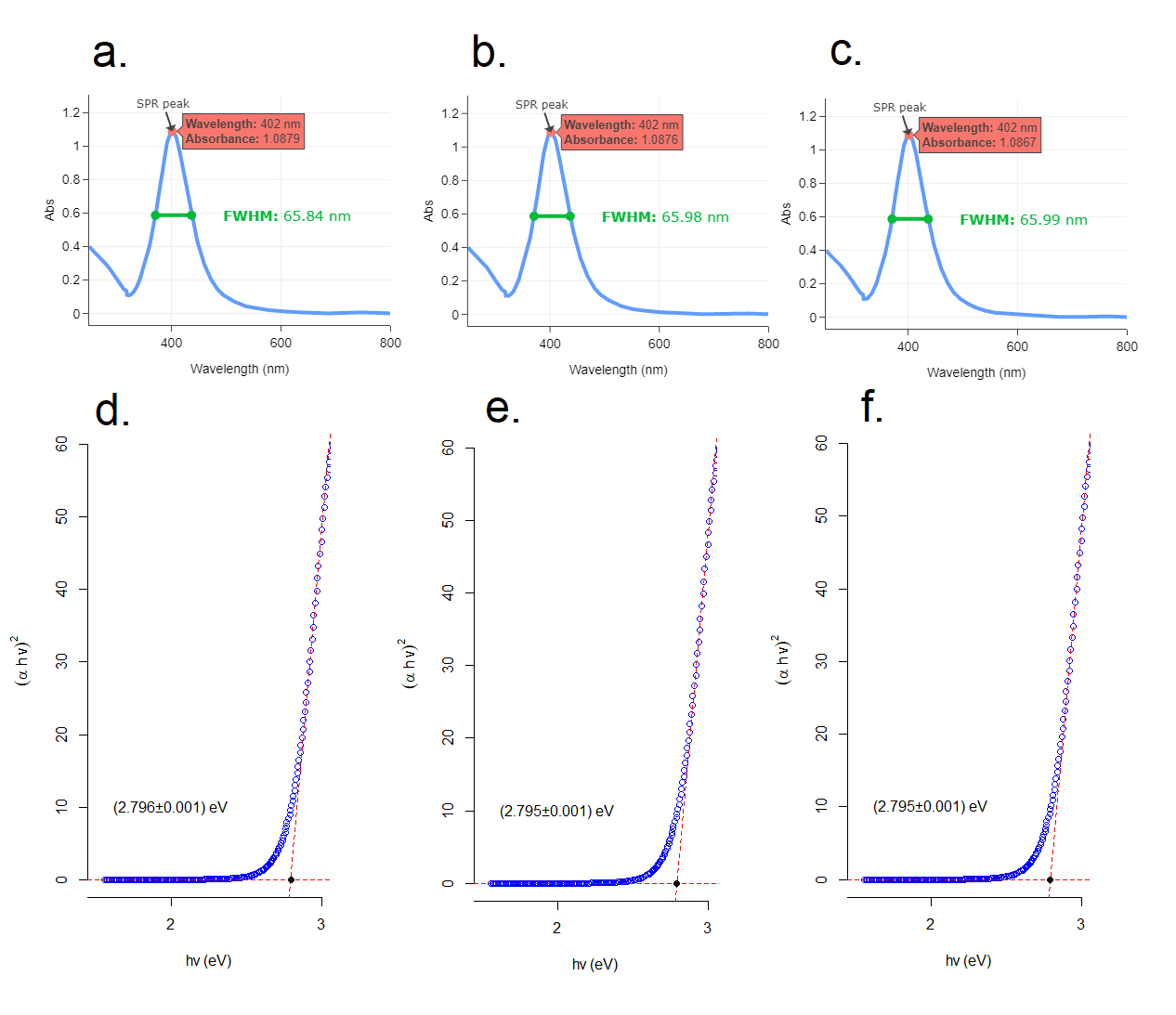


**Figure S8.** Optical properties (**a, b, c**) determined using [1] and bandgap energy of synthesized sub-15 nm AgNPs (**d, e, f**).

**
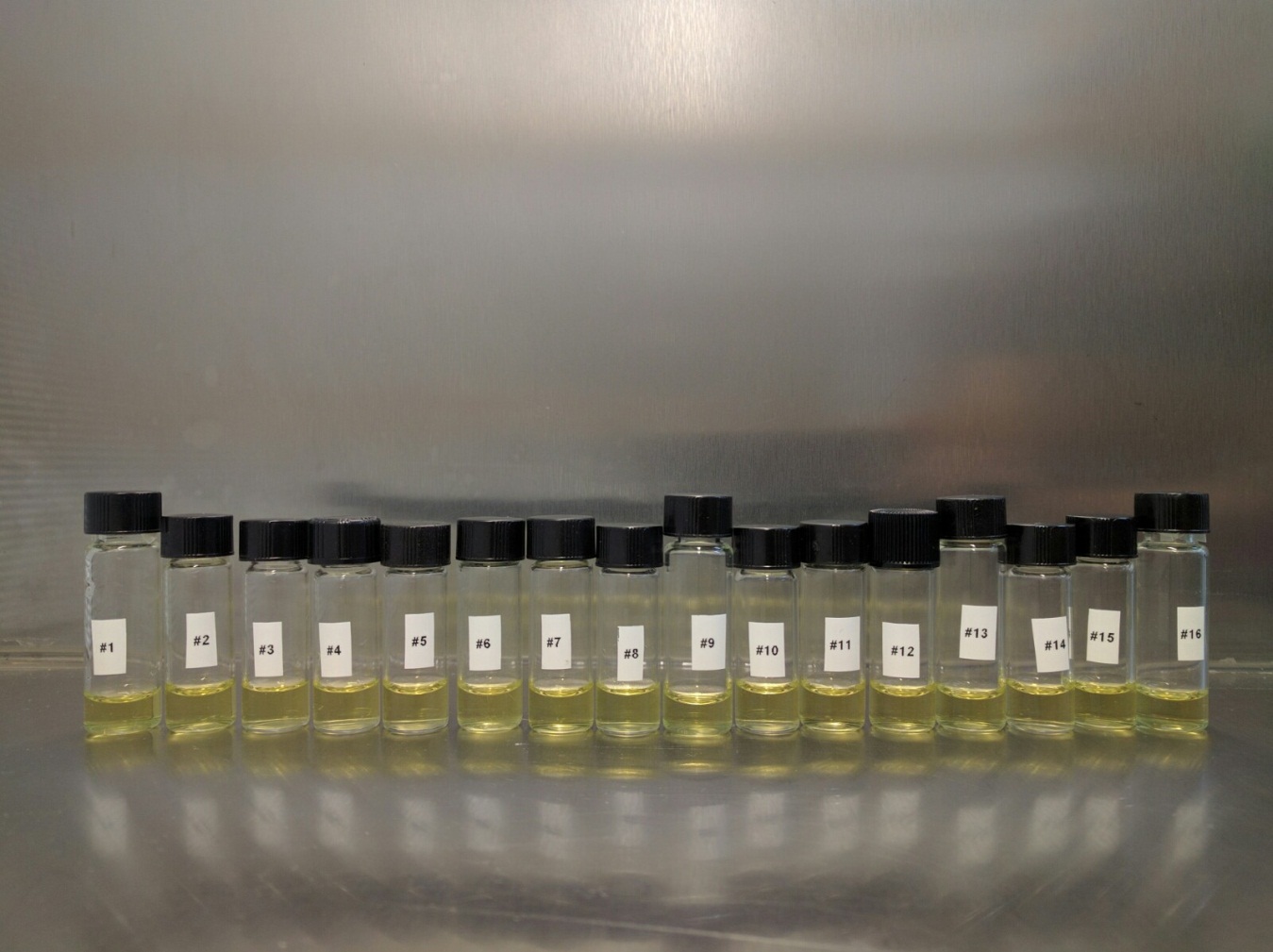
**

**Figure S9** Images of the subsamples generated to perform the stability studies of the AgNPs.

**Table S1.** Statistical summary of the particle size (nm) measurements made by AFM during the evolution of the synthesis of the AgNPs by sonochemistry.

| **Reaction time**  **(min)** | **Min.** | **Q_1_** | **Median** | **Mean** | **Q_3_** | **Max.** |
| --- | --- | --- | --- | --- | --- | --- |
| **4** | 5.9 | 6.7 | 16.0 | 12.3 | 16.5 | 72.4 |
| **16** | 1.1 | 4.3 | 6.4 | 8.5 | 10.0 | 44.6 |
| **92** | 3.4 | 8.7 | 9.8 | 10.0 | 10.7 | 76.3 |
| **140** | 4.9 | 8.4 | 9.1 | 9.4 | 10.4 | 18.1 |

**Table S2.** Equations involved in the determination of the bandgap energy (E_g_).

| **Equation** | **Description** | **Reference** |
| --- | --- | --- |
| $\left( \alpha h\nu\right)^{n}=K(h\nu-E_{g})$ | Tauc plot | [2] |
| $E= h\nu=\frac{1240}{\lambda}$ | Einstein’s photon energy | [3] |
| $I= I_{0}e^{-\alpha l}$ | Beer-Lambert law | [4] |
| $A=\log\frac{I_{0}}{I}$ | Absorbance | [4] |
| $\alpha=ln10 \frac{A}{l}$ | Absorption coefficient | [4] |

**Table S3.** Estimation of the bandgap energy (mean).

| **Description** | **Symbol** | **S_1_** | **S_2_** | **S_3_** |
| --- | --- | --- | --- | --- |
| **Correlation coefficient** | r^2^ | 1,00 | 1,00 | 1,00 |
| **Slope** | A_x_ | 233,1 | 232,8 | 232,4 |
| **Intercept** | B_x_ | -651,5 | -650,7 | -649,5 |
| **Band gap (eV)** | E_g_ | 2,796 | 2,795 | 2,795 |

**S_1_**: Sample 1. **S_2_**: Sample 2. **S_3_**: Sample 3.

**Table S4.** Expanded Measurement uncertainty of the bandgap energy

| **Description** | **Symbol** | **Value (eV)** |
| --- | --- | --- |
| **^a^Uncertainty Type A** | $u_{A}$ | 0,0002 |
| **^b^Uncertainty Type B (OLS)** | $u_{B}$ | 0,001 |
| **^c^Expanded Uncertainty (k=2)** | $U_{E_{g}}$ | 0,002 |
| **Conduction bandgap (Mean)** | E_g_ | 2,795 |

^a^Uncertainty Type A: $u_{A}=\frac{s}{\sqrt{n}}$.. ^b^Uncertainty Type B (OLS): $u_{B}= \frac{s_{xy}}{A_{x}}\sqrt{\frac{1}{p}+\frac{1}{N}+\frac{\left( \bar{x}_{0}-\bar{x}_{p} \right)^{2}}{Q_{xx}}}.$

^c^Expanded Uncertainty: $U_{E_{g}}=k\cdot u_{c} =2\cdot\sqrt{u_{A}^{2}+ u_{B}^{2}}$.

**References**

1. Calderón-Jiménez, B.; Sarmanho, G.; Murphy, K. E.; Montoro-Bustos, A. R.; Vega-Baudrit, J. NanoUV-VIS: An Interactive Visualization Tool for Monitoring the Evolution of Optical Properties of Nanoparticles Throughout Synthesis Reaction, *J. Res. Natl. Inst. Stan*. **122**, DOI: https://doi.org/[10.6028/jres.122.037](https://doi.org/10.6028/jres.122.037) (2017).
2. Makuła, P., Pacia, M., & Macyk, W. How to correctly determine the band gap energy of modified semiconductor photocatalysts based on UV–Vis spectra. J. Phys. Chem. Lett, 9, 6814–6817. DOI: <https://doi.org/10.1021/acs.jpclett.8b02892> (2018).
3. Braslavsky, S. E. Glossary of terms used in photochemistry, (IUPAC Recommendations 2006). *Pure Appl Chem*, 79, 293-465. DOI: <https://doi.org/10.1351/pac200779030293> (2007).
4. Gharibshahi, L., Saion, E., Gharibshahi, E., Shaari, A. H., & Matori, K. A. Structural and optical properties of Ag nanoparticles synthesized by thermal treatment method. *Materials*, 10, 402. DOI: <https://doi.org/10.3390/ma10040402> (2017).
